# Twisted 1‐ and 2‐Azaperopyrenes: Synthesis, Structure, and Properties

**DOI:** 10.1002/chem.202503136

**Published:** 2025-12-31

**Authors:** Ricardo Molenda, Arpine Vardanyan, Alexander Villinger, Peter Ehlers, Peter Langer

**Affiliations:** ^1^ Institute of Chemistry University of Rostock Rostock Germany

**Keywords:** arenes, aromaticity, catalysis, heterocycles, palladium

## Abstract

A synthesis of hitherto unknown 1‐ and 2‐azaperopyrenes, twisted nitrogen‐doped peropyrenes, is reported. The synthesis is based on a Brønsted acid‐mediated benzannulation of alkynes. Key alkyne intermediates are synthesized via two complementary routes, including a Pd/C‐catalyzed, copper‐ and amine‐free Sonogashira‐type coupling of (hetero)aryl chlorides, enabling access to both carbo‐ and heteroatom‐substituted peropyrene frameworks. X‐ray crystallography reveals twisted conformations with *bay* and end‐to‐end twist angles ranging between 10.0–19.9° and 5.5–25.0°, respectively, as well as unprecedented conformational isomorphism. The influence of regioselective single N‐doping within the twisted peropyrene framework on the photophysical and electrochemical properties were elucidated by combined experimental and computational studies and compared with those of the carbon congener.

## 1 Introduction

Nanographenes (NGs), defined as discrete molecular segments of graphene, are large polycyclic aromatic hydrocarbons (PAHs) with sizes of 1–100 nm [1–4]. Owing to their unique structures, they have been identified as promising platforms for next‐generation optoelectronics. While their size and edge topology are important determinants, heteroatom doping [5, 6] and contortion [7, 8] of the π‐skeleton exert a decisive influence on their optical and electronic properties, solid state stacking and molecular assembly. Peropyrene (**PP**), an armchair‐edged NG and higher homolog of pyrene, has gained considerable attention due to its intriguing properties such as high fluorescence quantum yield, high photostability, two‐photon absorption features, triplet‐triplet annihilation photon upconversion (TTA‐UC), and efficient charge transport [9, 10, 11, 12]. Peropyrene has also been explored as a potential singlet fission (SF) material, although its performance remains limited [10, 11]. Expanding the synthetic diversity of the peropyrene scaffold is highly desirable for tailoring its optoelectronic properties, with substitution at the *bay* regions (5,6,12,13‐positions) being particularly attractive, as it introduces significant steric congestion that gives rise to twisted, nonplanar conformations. However, the synthesis of *bay*‐substituted peropyrenes and their derivatives [13] is still scarce. The most efficient approaches involve two‐ or fourfold Brønsted acid‐mediated [14, 15, 16] or InCl_3_‐catalyzed [17, 18] benzannulations of alkynes. The Brønsted acid‐mediated alkyne annulation, however, has been shown to be limited to electron‐rich arylalkynes [17]. Additional strategies have recently emerged from radical chemistry of alkynes [19] and substituted phenalenyl derivatives [20].

**FIGURE 1:**
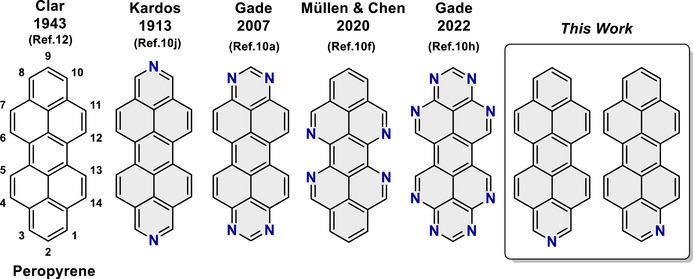
Chemical structure of peropyrene with atom numbering, and previously reported azaperopyrenes.

Nitrogen (N) doping of the PP framework offers further avenues for property modulation, as demonstrated by recent developments in functional azaperopyrenes (Figure 1) [21, 22, 23, 24, 25, 26, 27, 28, 29, 30]. However, the synthesis of twisted N‐doped peropyrenes remains rare, with only two examples [31, 32] reported since Clar's original synthesis of peropyrene in the early 1940s [33]. The synthesis of single‐N‐doped peropyrenes has, to the best of our knowledge, not been reported to date. Herein, we present a straightforward and efficient approach to *bay* substituted 1‐ and 2‐azaperopyrenes via Brønsted acid‐mediated alkyne benzannulation. In addition, we report the application of our protocol to the synthesis of a new peropyrene derivative with a related *bay*‐substitution pattern. The nonplanar (twisted) structures of the (aza)peropyrenes and their physical properties were investigated in detail using experimental and computational methods.

## 2 Results and Discussion

### 2.1 Synthesis

**SCHEME 1:**
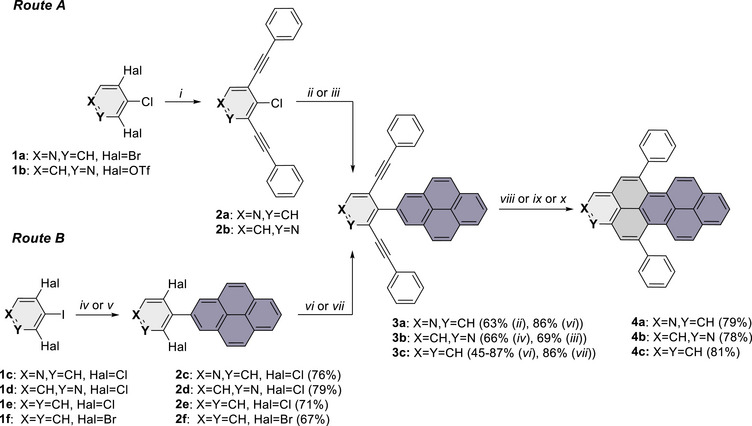
Synthetic route toward (aza)peropyrenes **4a**–**c**. Reagents and conditions: (*i*) PhC≡CH, Pd(PPh)_4_, CuI, HN*^i^* Pr_2_, MeCN, rt‐50 °C. (*ii*) 2‐(Bpin)pyrene, Pd(PPh)_4_, K_3_PO_4_, dioxane/H_2_O, 100 °C (**2a**). (*iii*) 2‐(Bpin)pyrene, PdCl_2_(MeCN)_2_, XPhos, K_3_PO_4_, dioxane/H_2_O, 100 °C (**2b**). (*iv*) 2‐(Bpin)pyrene, Pd(PPh)_4_, K_3_PO_4_, dioxane/H_2_O, 90 °C (**1c,d**). (*v*) 2‐(Bpin)pyrene, Pd(PPh)_4_, K_2_CO_3_, THF/H_2_O, 80 °C (**1e,f**). (*vi*) PhC≡CH, Pd/C, XPhos, K_2_CO_3_, DMF, 110 °C (**2e,f**). (*vii*) PhC≡CH, Pd(OAc)_2_, XPhos, CuI, HN*^i^* Pr_2_, toluene, 100 °C (**2f)**. (*viii*) *p*TsOH, 100 °C (**3a**). (*ix*) TfOH, CH_2_Cl_2_, 0‐rt (**3b**). (*x*) InCl_3_, toluene, 100 °C (**3c**).

Our study started with the retrosynthetic analysis for the 1‐ and 2‐azaperopyrene precursors **3a** and **3b**. Two synthetic routes were identified starting either from dialkynylpyridines **2a,b** or dichloropyridylpyrenes **2c,d** (Scheme 1). Route A is based on our previous protocol for the synthesis of 1‐ and 2‐azapyrenes (Scheme 1) [34, 35]. Accordingly, bis(phenylethynyl)pyridine **2a** was subjected to Suzuki coupling with pyrene pinacol boronate ester (2‐(Bpin)pyrene) using Pd(PPh_3_)_4_ as catalyst, K_3_PO_4_ as base and 1,4‐dioxane as solvent. However, under these conditions, significant amounts of an unidentified side product of similar polarity were formed, as observed in all other test reactions (Table S1). Consequently, purification of the cross‐coupling product proved to be a major challenge, and all attempts using standard chromatographic methods failed to achieve complete separation. Finally, careful washing of the chromatographically prepurified residue with small amounts of methanol afforded the 2‐azaperopyrene (2‐APP) precursor **3a** as a pure compound in 63% yield. In contrast, no side‐product formation was observed in the analogous synthesis of the 1‐azaperopyrene (1‐APP) precursor **3b**, which was obtained from **2b** in 69% yield after optimization (Table S2).

We further investigated the reverse order of cross‐coupling reactions (Scheme 1, route B). Suzuki coupling of 2‐(Bpin)pyrene with commercially available 3,5‐dichloro‐4‐iodopyridine **1c** furnished intermediate **2c** in good yield. However, subsequent Sonogashira coupling of **2c** with phenylacetylene to give 2‐APP precursor **3a** under homogeneous conditions proved to be unsuccessful under various combinations of Pd catalysts and phosphine ligands, as summarized in Table S3. Finally, we tested a slightly modified copper‐ and amine‐free Pd/C protocol using XPhos as ligand and K_2_CO_3_ as base [36]. Under these conditions, **2c** underwent clean conversion with phenylacetylene within 2 h, affording **3a** in an excellent yield of 86%. Encouraged by this result, the same strategy was subsequently applied to synthesize 1‐APP precursor **3b**, which was readily obtained from **2d** and phenylacetylene in a yield comparable to route A.

To further demonstrate the versatility, we also applied the same strategy to the synthesis of an all‐carbon peropyrene (PP) intermediate, which offers a valuable and practicable alternative to previously reported routes that rely on presynthesized bis(arylalkynyl)phenylboronates and 2‐bromopyrenes [14, 16, 17]. Accordingly, **2e** was prepared from 1,3‐dichloro‐2‐iodobenzene **1f** and 2‐(Bpin)pyrene in 71% yield. Subsequent reaction with phenylacetylene using Pd/C conditions, afforded the PP precursor **3c** within 2 h in a high yield of 87%. Our approach thus enables straightforward access to both PP and APP precursors starting from less expensive, commercially available (hetero)aryl chlorides. Noteworthy, replacing dichloride **2e** by dibromide **2f** afforded only a moderate yield of **3c** (45%) after 24 h. In contrast, employment of homogenous conditions (Pd(OAc)_2_/XPhos) allowed the synthesis of **3c** from **2f** in only 1 h and in a high yield of 86% which is comparable to that observed during the reaction of **2e** under heterogeneous Pd/C conditions. The better yield in case of chloride as compared to bromide is unusual and might be explained by formation of side‐products in case of the bromide which could, however, not be isolated or detected by TLC.

The final benzannulation was initially investigated using the 2‐APP precursor **3a**. Various Brønsted acids, that is, methanesulfonic acid (MsOH), *p*‐toluenesulfonic acid monohydrate (*p*TsOH·H_2_O), and trifluoromethanesulfonic acid (TfOH) were screened, and the results are summarized in Table S4. In general, all acids proved suitable for promoting the twofold benzannulation. The best yield was obtained in neat *p*TsOH·H_2_O, affording **4a** in 79% yield. TfOH in anhydrous CH_2_Cl_2_, under an inert atmosphere, proved more efficient for the benzannulation of 1‐APP precursor **3b**, providing the corresponding 1‐azaperopyrene **4b** within 3 h in 78% yield. The reaction of **3b** using *p*TsOH·H_2_O resulted in incomplete cyclization and a mixture of **4b** and the respective monocyclized species were obtained after 24 h. Attempts to cyclize **3c** to all‐carbon peropyrene **4c** under the same Brønsted acid conditions proved to be unsuccessful, highlighting the key role of nitrogen in promoting the acid‐mediated cyclization. As expected, π‐Lewis acid mediated benzannulation of **3c** with indium(III) chloride (InCl_3_) proceeded smoothly to furnish hitherto unknown peropyrene **4c** in 87% yield.

### 2.2 Single‐Crystal X‐Ray Diffraction Analysis

**FIGURE 2:**
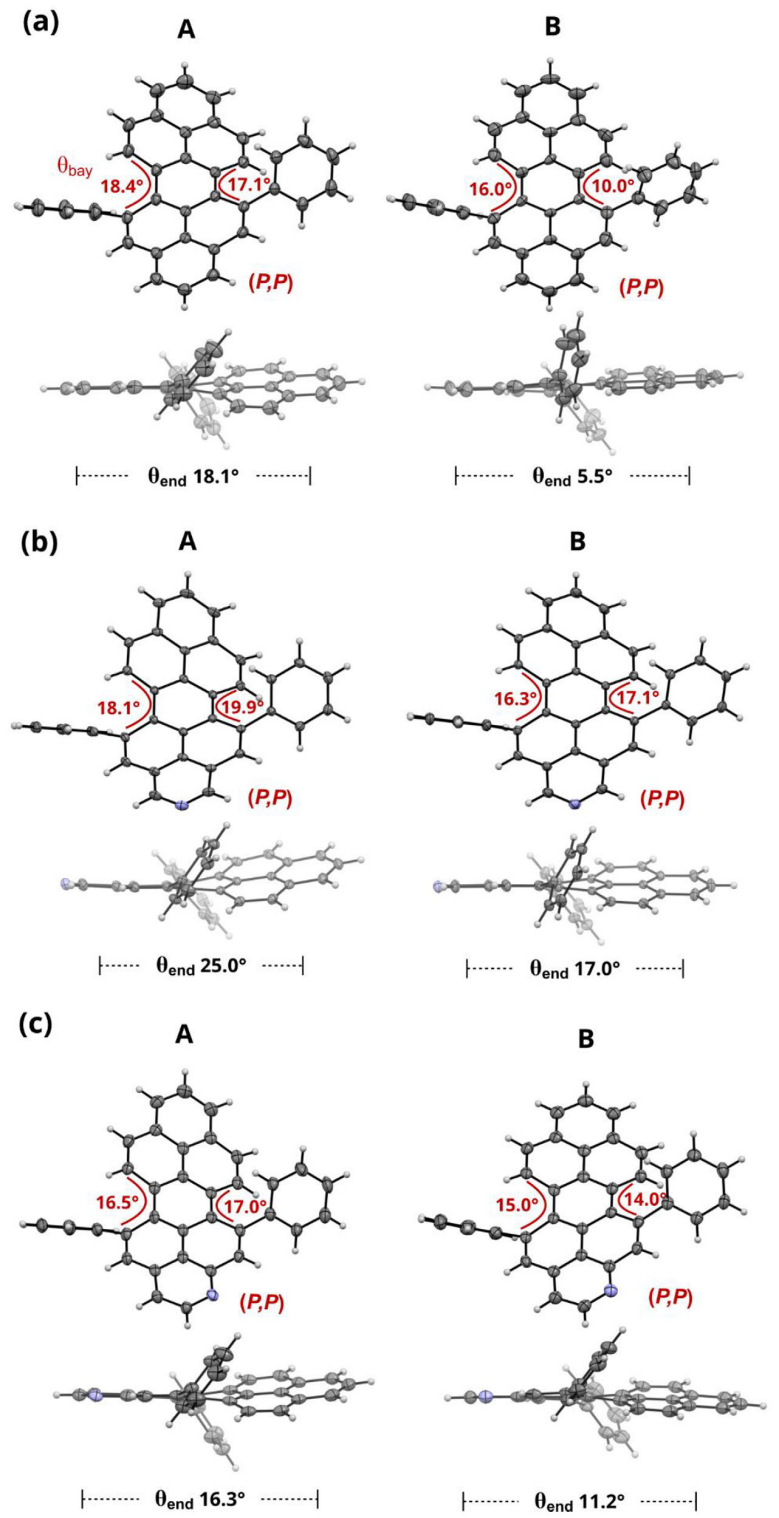
Top and side views of the X‐ray structures with *bay* (*θ*_bay_) and end‐to‐end (*θ*_end_) twist angles of (a) **4c** (b) **4a** and (c) **4b**. A and B denote two symmetry‐independent conformations. Only the (*P*,*P*)‐enantiomers are shown. Thermal ellipsoids are drawn at the 50% probability level. Color code: C (grey), N (blue).

The structures of **4a**–**c** were independently confirmed by single‐crystal X‐ray diffraction. Suitable crystals were grown from CH_2_Cl_2_/EtOAc solutions at room temperature, allowing for a direct comparison of the N‐doping effect on the molecular structures and crystal packing arrangements under the same crystallization conditions. In line with previously reported 5,13‐*bay*‐arylated PPs [14, 16, 17], all peropyrenes crystallize in twisted conformations, with both (*P*,*P*)‐ and (*M*,*M*)‐configurations in the crystal packing. Although these peropyrenes are expected to exist as a pair of enantiomers and an achiral conformer with low interconversion barriers (*vide infra*), no *meso* (*P*,*M*)‐isomers were observed in any of the crystal packing arrangements. Instead, a second symmetry‐independent molecule with ideal positional disorder (50:50) creating local inversion symmetry is present in the crystal structure of **4c**. In the asymmetric unit, this structure is represented in the (*P*,*P*)‐configuration (Figure 2a, structure B), indicative of conformational isomorphism. This is unambiguously revealed in the crystal packings of **4a** and **4b**, where two symmetry‐independent pairs of (*P*,*P*)/(*M*,*M*) enantiomers coexist in the same crystal structure (Figure 2b,c).

Such packing, in which four chiral stereoisomers cocrystallize within a single crystal, is unprecedented for 5,13‐*bay*‐substituted peropyrenes, and related observations have only scarcely been reported [37, 38]. However, such structural disorder, which is of high importance for material applications, often seem to be unrecognized in the literature as we identified the same phenomenon in several other reported crystal structures [17, 19, 39, 40, 41, 42], including a single *bay*‐substituted peropyrene [19].

**FIGURE 3:**
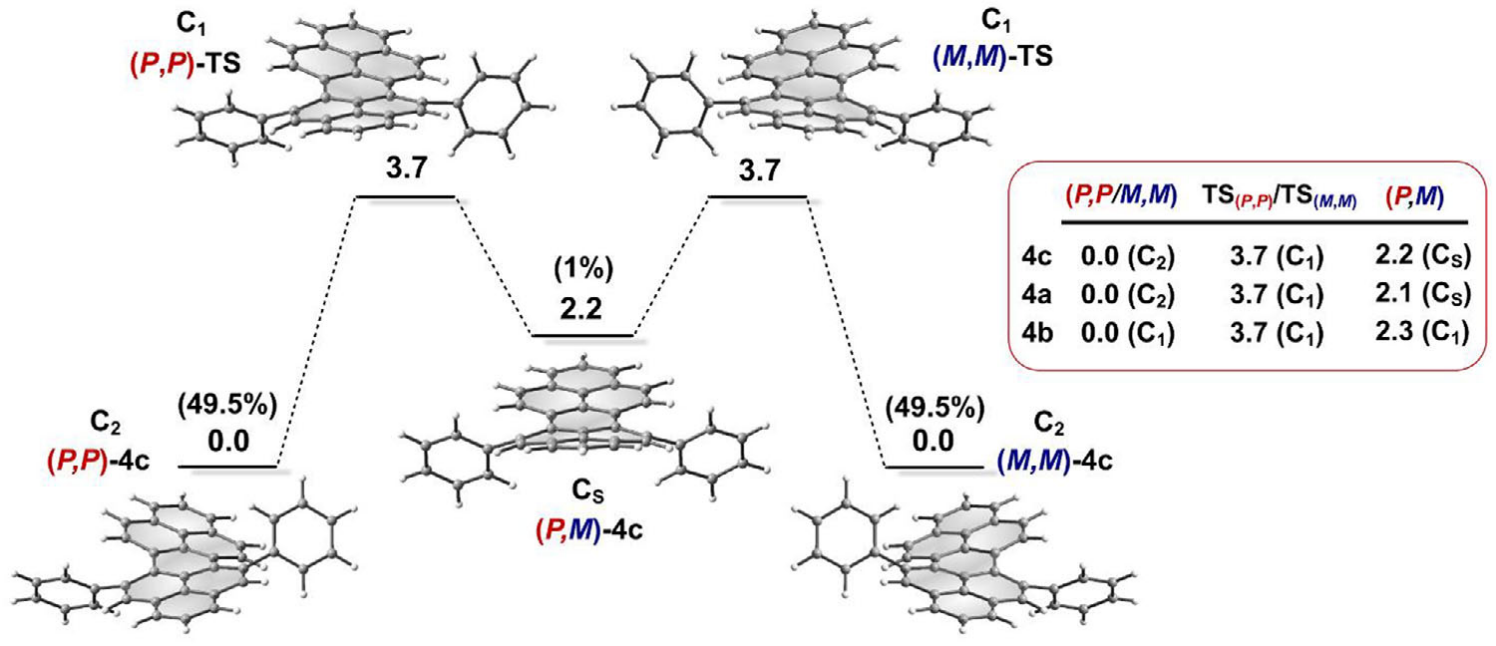
Interconversion pathway with relative Gibbs free energies (Δ*G*, kcal mol^−1^) and Boltzmann conformational populations at 298 K, calculated at the B3LYP‐D3(BJ)/6‐311G(d,p) level of theory.

To gain insight into the conformational selectivity and elucidate the configurational stability, the interconversion of **4a**–**c** was calculated (Figure 3). Following the proposed two‐step mechanism [15, 32], rotation of one phenyl group in (*P,P*) or (*M,M*) provides the *meso*‐(*P,M*) conformer, which either reverts to the original configuration or undergoes a second inversion by rotation of the other phenyl group to complete the enantiomerization.

The twisted enantiomers with either C_2_‐ (**4a,c**) or C_1_‐ (**4b**) symmetry are thermodynamically more stable than the achiral C_S_‐ (**4a,c**) or C_1_‐ (**4b**) symmetric (*P*,*M*)‐conformers by about 2 kcal mol^−1^. Consequently, the *meso* isomers are expected to constitute only about 1% of the population at room temperature, with the enantiomers collectively accounting for the remaining 99%. This distribution is consistent with the absence of (*P*,*M*)‐conformations in the crystal structures. The activation free energies Δ*G* ^‡^(298 K) for the chiral‐to‐*meso* interconversion via the C_1_‐symmetric transition states are calculated to be 3.7 kcal·mol^−1^ for all peropyrenes, corresponding to enantiomer half‐lives of approximately 115 ps, as determined using the Eyring equation (transmission coefficient *κ*  =  0.5). These low interconversion barriers emphasize the high configurational flexibility of PPs bearing one aryl substituent in each *bay* region, which remains unaffected by single N‐doping of the peropyrene backbone. Consequently, compared to tetra‐*bay*‐arylated PPs [15, 18] (Δ*G* ^‡^ ≈ 29 kcal mol^−1^), the enantiomers of the studied (A)PPs rapidly interconvert in solution at room temperature and are only resolved in the solid state, consistent with the sharp signals observed in the ^1^H NMR spectra.

Beyond the requirement for configurational stability, structural flexibility can promote favorable packing and enhance electronic coupling, both of which are critical for material applications [43]. The conformational flexibility of 5,13‐*bay*‐arylated (A)PPs is also reflected in the large variations observed for the *bay* (*θ*_bay_) and end‐to‐end (*θ*_end_) twist angles (Figure 3; definitions in Figure S1), ranging from 10.0–19.9° and 5.5–25.0°, respectively, as well as in the nonuniform twisting between the two *bay* regions, with deviations of 0.5–6°. Similar trends are shown in previously reported X‐ray crystal structures of 5,13‐substituted PPs with *bay* torsion angles between 13.5–19.9°, end‐to‐end twists between 12.8–22.9° and deviations of 0.1–3.2° between the two *bay* regions [14, 16, 17]. This modulation in conformational distortion is attributed to crystal packing effects, as further corroborated by DFT (B3LYP‐D3(BJ)/6‐311G(d,p)) optimized gas‐phase structures, showing comparable *bay* (∼19°) and end‐to‐end (∼20°) twist angles, along with uniform twisting across both *bay* regions (Table S6).

### 2.3 Bond‐Lengths Analysis, Electron Delocalization, and Aromaticity

**FIGURE 4:**
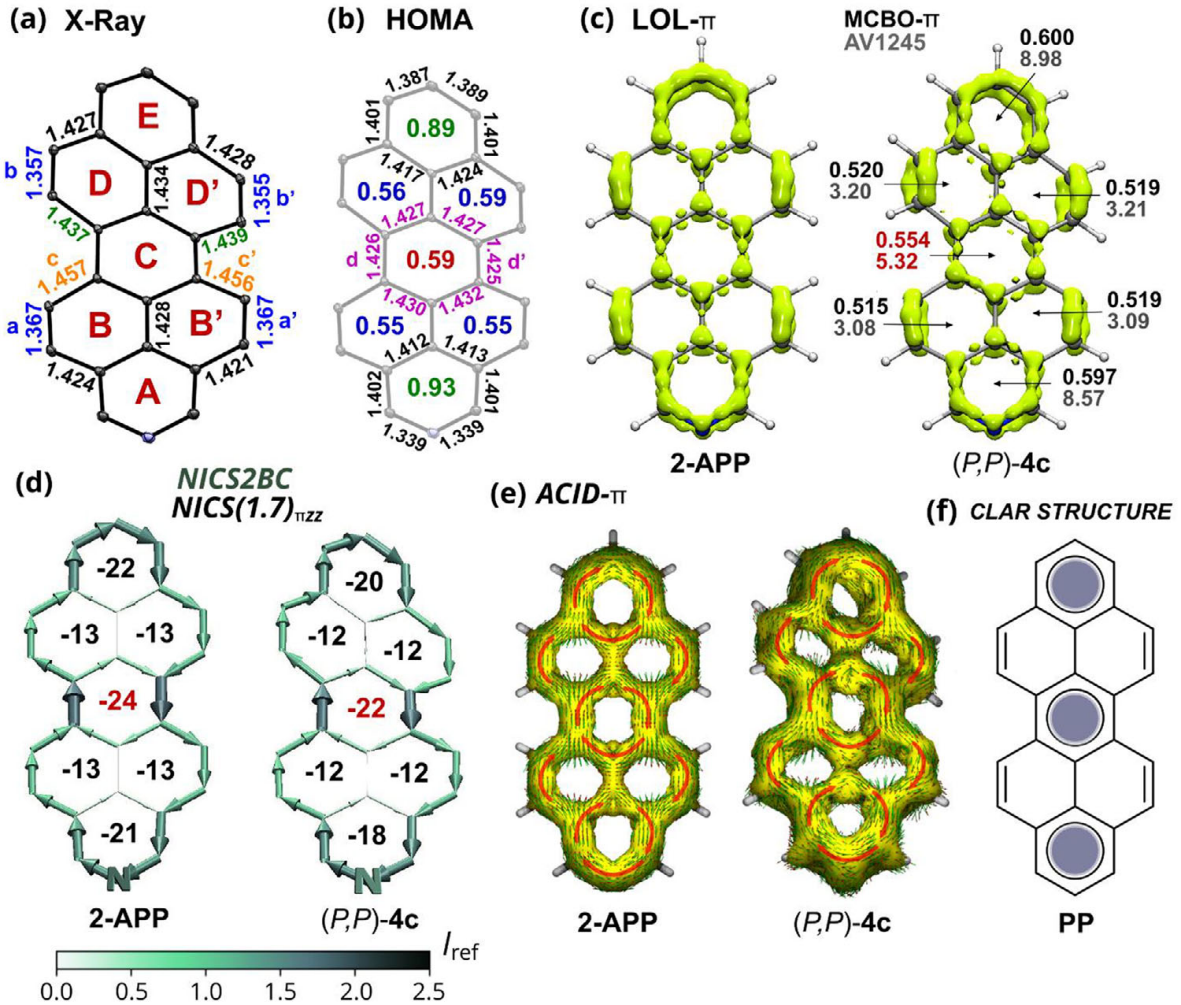
Aromaticity analysis exemplified on (*P*,*P*)‐**4a** and pristine planar 2‐azaperopyrene (**2‐APP**). Peripheral phenyl substituents of (*P*,*P*)‐**4a** are not shown. (a) X‐ray structure of (*P*,*P*)‐**4a** (conformer B) with selected bond length (Å). (b) HOMA values with bond lengths (Å) of rings A, C, and E based on the X‐ray crystal structure of (*P*,*P*)‐**4a** (conformer B). (c) LOL‐π maps (isovalue 0.55), MCBO‐π and AV1245 values. (d) NICS2BC bond current graphs and NICS(1.7)_πzz_ values. Bond current strength is reported relative to *I*_ref_ (bond current of benzene, 11.5 nAT^−1^). (e) ACID‐π plots (isovalue 0.03). (f) Clar structure of peropyrene (**PP**).

Peropyrene (**PP**) exhibits characteristic bond length features of pyrene [44], and the general bond length pattern of **PP** is essentially unaltered by the twist operation and single N‐doping, as revealed by bond length analysis. Accordingly, bonds a,a’ and b,b’ at the edges of the four K‐regions indicate the typical double bond character with bond lengths of 1.340–1.369 Å (Figure 4a; Table S7). The corresponding bonds connecting each of these K‐region edges range between 1.415–1.471 Å, with the C─C bonds (c/c’) in the sterically crowded *bay* regions being significantly elongated (1.454–1.471 Å), comparable to bond lengths shown in reported X‐ray structures of related 5,13‐*bay*‐arylated PPs [14, 16, 17] (1.453–1.462 Å). Along the molecular long axis, rings A and E exhibit typical aromatic bond lengths, whereas the central ring C shows comparatively longer bonds, indicative of a reduced aromatic character. The average bond length of the central ring, determined across all structures, is 1.426 Å and remains essentially unchanged compared to planar peropyrenes [10, 11] (1.424 Å; Table S8). This indicates that the twist operation does not significantly perturb the aromatic character of the central ring. In contrast to the single‐bond character in perylene [45], the C─C bonds (d/d’) connecting the two formal (aza)phenalenyl radical fragments to form peropyrene exhibit an average length of 1.42 Å (Figure 4b; Table S7). The absence of bond length alternation further underscores the presence of effective π‐electron delocalization across the central ring (Table S8).

However, the aromaticity of the central ring and its depiction by the Clar structure (Figure 4f) have been the subject of debate. Gutman and Agranat argued that the central ring of **PP** is neither “aromatic” nor empty according to Clar's classification and that a three‐sextet Clar structure does not adequately describe its π‐electron distribution based on Hückel‐type (ef‐model) calculations [46]. This view was later challenged by ab initio DFT calculations, which predicted that the central ring features longer C─C bonds than expected for a Clar sextet, but with negligible bond‐length alternation [47]. These predictions were subsequently confirmed by the single‐crystal X‐ray structure of **PP** and related derivatives, including those of the present study. Randić, combining ring‐bond orders and variations in Pauling bond orders, concluded that **PP** follows the Clar model, though the central ring is less aromatic than the terminal ones [48]. Hückel–London–Pople–McWeeny (HLPM) topological π‐electron ring current calculations by Mallion [49, 50] predicted a long time ago, that the central ring possesses the highest ring current intensity, participating the most in the overall conjugation among the individual rings in **PP**.

These findings emphasize that determination of the aromatic features of peropyrene is not as straightforward as it appears at first glance. This calls into question the aromaticity predictions by widely used aromaticity indices, such as the harmonic oscillator model of aromaticity (HOMA) and nucleus‐independent chemical shift (NICS). In this context, it is surprising that among the published studies on (aza)peropyrenes, only one study reports nonrecommended isotropic NICS [51] values for a twisted dicyclopenta‐fused peropyrene, while no study has applied HOMA [13]. To fill this gap, we evaluated both metrics for the planar and twisted structures.

The overall trend in HOMA [52] values (parameters: *R*_CC_,_opt_  =  1.388 Å, *α*_CC_  =  257.7 Å^−2^; *R*_CN,opt_  =  1.334, *α*_CN_  =  93.52 Å^−2^), derived from both crystallographic and calculated structures, indicates that the central ring C is less aromatic than rings A and E (Figure 4b and Tables S9,10). The same trend is observed using the newly parametrized HOMAc (parameters: *R*_CC,opt_ = 1.392 Å, *α*_CC_  =  153.37 Å^−2^; *R*_CN,opt_ = 1.333, *α*_CN_  =  111.83 Å^−2^) index (Tables S9,10) [53]. While rings B/B′ and D/D′ generally show lower HOMA values than ring C in the crystal structures, the differences are relatively small and further decrease in the calculated gas‐phase structures, indicating that packing‐induced conformational distortions contribute to aromaticity differences in the solid state (Table S11). A different interpretation emerges from the NICS(1.7) and NICS(1.7)_πzz_ values, indicating that the central ring C is slightly more aromatic than rings A and E, while rings B/B′ and D/D′ remain distinctly less aromatic (Figure 4d, Tables S12,13). This trend remains essentially unchanged between gas‐phase optimized and crystal geometries.

Electron delocalization pathways visualized by anisotropy of induced current density [54] (ACID)‐π maps and NICS2BC [55] bond current graphs (Figures 4d,e and S2,3) align with previously reported HLMP topological bond current [56], ring current (RC), and multicenter bond indices ring current (MCBI‐RC) maps [57] for pristine **PP**. Accordingly, a clockwise (diatropic) current is observed circulating around the perimeter, accompanied by two semi‐global diatropic (aza)pyrenic currents (A,B,B′,C and C,D,D′,E) that encompass the central ring, as well as three local diatropic current circuits within the long‐axis rings (A,C, and E). Similar to HLPM [49, 50] topological ring current calculations, NICS2BC predicts the central ring (C) with the highest ring current strength, as expressed by the ring weight value (Figure S2). The K‐region rings B/B' and D/D' show branched circuits and, therefore, do not form local currents, emphasizing their role as bridges in the formation of the (semi)global currents. This aligns with the lower aromatic character of these rings according to the bond length pattern, less negative NICS and lower HOMA values.

The electron delocalization measure of aromaticity, given by the localized orbital locator [58] maps of the π‐orbitals (LOL‐π), also reveal stronger six‐center π‐electron conjugation in rings A, C, and E as compared to rings B/B’ and D/D’ (Figure 4c). This is reflected by the partially disconnected LOL‐π isosurfaces (see Figure S4 for different isovalues). The π‐orbitals of rings B/B' and D/D' are primarily located at the four C─C bonds of the K‐region edges, which agrees with the olefinic character according to the crystallographic analysis. The chosen isosurface representation in Figure 4c further highlights the differing degrees of π‐electron delocalization across rings A, C, and E, with rings A and E exhibiting more pronounced six‐center delocalization than the central ring. This is further supported by the multicenter bond order [59, 60] (MCBO‐π) and the average of the 4‐center multicenter indices (AV1245 [61]) values for the transferability of the delocalized electrons (Figure 4c and Tables S14,15). The AV1245 analysis further predict that π‐electron delocalization along the (aza)pyrene subunits is slightly more favored than delocalization along the perimeter, with the relative preference between these pathways modulated by the N‐doping position (Figure S6 and Table S16).

The combined results from the aromaticity analyses thus inferred that the π‐electron structure of peropyrene follows the Clar model (Figure 4f) with three sextets of higher (rings A and E) and lower (ring C) aromatic character, consistent with Randić’s [48] conclusion from bond order analysis. The *bay* substitution induced twist operation and the presence of N‐atoms in the investigated (A)PPs do not significantly alter the overall electron delocalization or aromaticity relative to the parent planar **PP**, as indicated by the applied aromaticity metrics. The most negative NICS value obtained for the central ring, which is associated with the strongest ring current, as predicted by HLPM and NICS2BC, may be rationalized by its construction from two non‐Kekulé phenalenyl radical fragments, whose fusion inherently induces a strong ring current in the newly formed six‐membered central ring, as outlined by Haigh and Mallion [62]. However, a higher current density does not automatically equate with increased (local) aromaticity, as suggested by the combined results of the aromaticity analysis.

Peropyrene thus serves as a representative example where HOMA and NICS predict different local trends in aromatic character within the π‐conjugated structure (central ring), with HOMA showing better agreement with other aromaticity indices, emphasizing the importance of using multiple aromaticity metrics when evaluating aromatic properties of π‐extended PAHs. This inconsistency between HOMA and NICS is also evident in higher peropyrene homologous [63], indicating that the inner sextets along the long molecular axis are consistently less aromatic than the terminal ones. This is supported by ring bond order analysis [48], LOL‐π maps (Figure S5), and X‐ray crystallographic bond length patterns [63, 64].

### 2.4 Photophysical Properties

**FIGURE 5:**
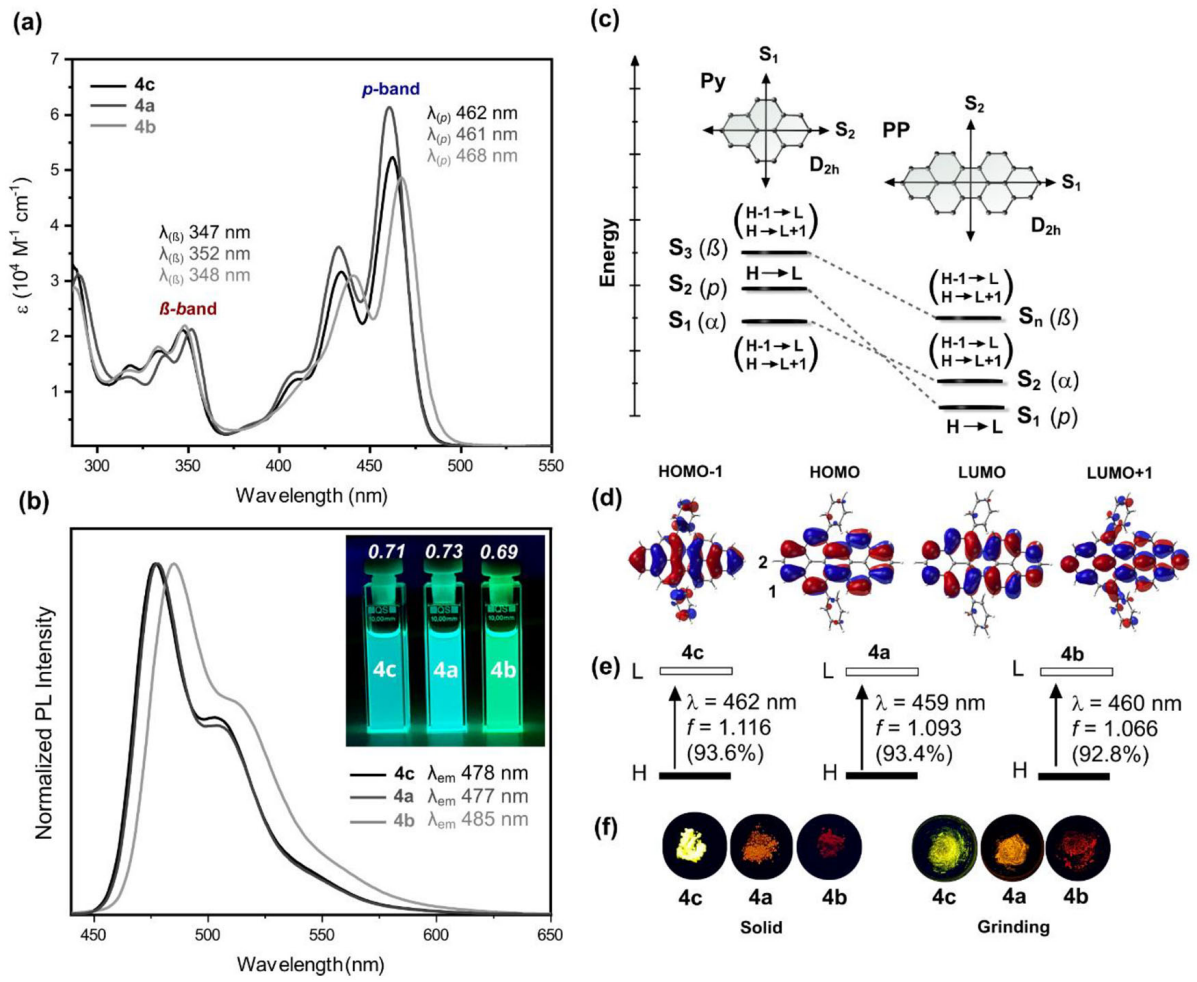
(a) UV‐vis absorption spectra of **4a–c** in toluene at 20 °C (c ≈ 1.0 × 10^−5^ M); absorption band names assigned using Clar notation. (b) Emission spectra (*λ*_ex_.  =  420 nm). Inset: images of dilute toluene solutions under UV light (*λ*  =  365 nm). (c) Energy diagram of excited states, orbital contributions, and corresponding Clar nomenclature (*α*,*p*,*ß*) of pyrene (**Py**) and peropyrene (**PP**). Transition dipole moment directions and molecular symmetry are also shown. (d) Molecular orbitals of (*P*,*P*)‐**4c** with selected atom numbering (B3LYP‐D3(BJ)/6‐311G(d,p); isovalue 0.02 a.u.). (e) TDA‐DFT (B3LYP‐D3(BJ)/6‐311+G(d,p))‐derived excitation energies, oscillator strengths (*f*), and percentage orbital contributions for the major electronic transitions of **4a–c**. (f) Solid state samples under UV light (*λ*  =  365 nm) before and after grinding.

Building on these structural and electronic insights, the optical properties of the N‐doped peropyrenes were investigated in comparison to **4c** with the photophysical data summarized in Table 1. As shown in Figure 5, the UV‐Vis absorption and emission spectra follow those of previously reported 5,13‐*bay*‐arylated PPs [14, 16, 17] and resemble the characteristic band intensity distribution and vibronic progression features of pristine **PP** [9, 10, 11]. Accordingly, the absorption spectra are dominated by the intense *p*‐absorption band corresponding to the long axis polarized dipole allowed S_1_ ← S_0_ transition from the highest occupied molecular orbital (HOMO) to the lowest unoccupied molecular orbital (LUMO) (Figure 5e, Tables S19,21). The α‐band of the optically dark state which corresponds to the S_1_ ← S_0_ transition in (aza)‐pyrenes [34, 35, 65], derived from the short axis polarized HOMO‐1 → LUMO and HOMO → LUMO+1 excitations, becomes the higher energy S_2_ ← S_0_ transition and is hidden under the intense *p*‐absorption band. Hence, 1D π‐extension from pyrene (**Py**) to **PP** leads to an inversion of the two lowest‐energy singlet excited states while preserving D_2h_ symmetry (Figure 5c). The close energetic proximity between the optically dark S_2_ and bright S_1_ state in **PP** has been demonstrated by two‐photon excitation spectroscopy, and its solvent‐polarity dependent mixing leads to unusual radiative decay rates and an almost twofold increase of the fluorescence lifetime from nonpolar to polar solvents [9, 10].

**TABLE 1:** Photophysical data of peropyrenes **4a–c** in toluene at 20 °C (c ≈ 1.0 × 10^−5^ M).

Compound	*λ*_abs_ (nm)	*ε* (10^4^ M^−1^ cm^−1^)	*λ*_em_ (nm)	Strokes shift (cm^−1^)	*Φ*_F_ ^a^	*τ* ^b^ (ns)	*τ*_0_ ^c^ (ns)	*k*_r_ ^d^ (10^7^ s^−1^)	*k*_nr_ ^e^ (10^7^ s^−1^)
4c	462	5.32	478	724	0.71	2.58	3.63	27.5	11.2
4a	461	6.14	477	728	0.73	2.80	3.83	26.1	9.6
4b	468	4.86	485	749	0.69	2.88	4.17	23.9	10.7

^a^ determined relative to coumarin 153 in ethanol (*Φ_F_*  =  0.4, *λ*_ex._  =  420 nm).

^b^measured in air.

^c^natural lifetime (*τ*/*Φ*_F_).

^d^radiative decay rate constant (*Φ*_F_/*τ*).

^e^nonradiative decay rate constant (1/*τ*−(*Φ*_F_ */τ*)).

Compared to parent planar **PP** [9, 10] (λ(*p*)  =  442 nm, in toluene), the *p*‐band absorption maximum of **4c** (λ(*p*)  =  462 nm) is redshifted by 20 nm. Nitrogen doping at the 2‐position has a negligible effect on the position of the *p*‐absorption band relative to **4c**, while nitrogen doping at the 1‐position induces a slight redshift of 6 nm for **4b** (λ(*p*)  =  468 nm). This trend can be rationalized in a similar way as in (aza)‐pyrenes [34, 35], by the spatial distribution of the HOMO and LUMO, which exhibit a node at the 2‐position and large orbital coefficients at the 1‐position (Figure 5d). The molar extinction coefficients (*ε*) at the *p*‐band maxima range from 4.86–6.14 × 10^4^ M^−1^ cm^−1^, with the highest value observed for **4a** and the lowest for **4b**.

Typically, the *p*‐absorption band exhibits a progressive redshift on the order of 100 nm upon π‐extension from pyrene to peropyrene and terropyrene [66]. This characteristic redshift is also observed between 1,3,6,8‐tetraphenylpyrene [67] (*λ*(*p*)  =  356 nm) and 1,3,8,10‐tetraphenylperopyrene [68] (*λ*(*p*)  =  456 nm), and likewise in the corresponding diaza analogues bearing nitrogen atoms at the nodal‐plane positions [27, 69]. Twisting of the PP backbone enhances this redshift to 115 nm, as revealed by comparing the *p*‐band maxima of K‐region substituted 4,10‐diarylpyrene (*λ*(*p*)  =  352 nm) and 5,13‐diarylperopyrene (*λ*(*p*)  =  467 nm) [14]. The same degree of redshift is shown in the 2‐aza series from 5,9‐diphenyl‐2‐azapyrene [34] (*λ*(*p*)  =  346 nm) to 2‐azaperopyrene **4a** and further increases to 125 nm from 5,9‐diphenyl‐1‐azapyrene [35] (*λ*(*p*)  =  343 nm) to 1‐azaperopyrene **4b**. These trends highlight the combined influence of backbone twisting and site‐specific nitrogen doping on the lowest‐energy electronic transition.

The influence of site‐specific N‐doping is further evident in the higher‐energy β‐band, which shows an opposite trend as compared to the *p*‐band, reflecting the different orbital characters and sensitivities of the underlying electronic transitions. Relative to **4c** (*λ*(ß) = 347 nm), the β‐band absorption maximum of **4a** is bathochromically shifted by 5 nm, while no noticeable shift is observed for **4b**. According to TDA‐DFT calculations (B3LYP‐D3(BJ)/6‐311G(d,p)), the β‐bands arise from HOMO‐*n* → LUMO and HOMO → LUMO+1 (*n*  =  1,2,3) transitions (Table S19,21). The HOMO‐*n* and LUMO+1 orbitals show large coefficients in the 2‐position (Figures 5d and S8) and, thus, these orbitals are directly influenced by substituents, which supports the experimental observation.

The fluorescence spectra mirror the *p*‐band absorptions and their associated redshifts, with emission maxima between 477 and 485 nm (Figure 5b). The Stokes shifts remain small (721–791 cm^−1^) in comparison to those of planar peropyrenes [11], emphasizing that twisting still imparts considerable rigidity, with small structural reorganization upon photoexcitation. Fluorescence lifetimes of **4c** (2.58 ns), **4a** (2.80 ns), and **4b** (2.88 ns) fall within the range reported for a twisted PP [14] bearing one aryl substituent in the *bay* region (*τ*  =  2.5 ns, in toluene) and are comparable to those of the parent **PP** [9, 10] (*τ*  =  2.58–3.0 ns in toluene). Slightly longer lifetimes (*τ*  =  3.14–3.40 ns in CH_2_Cl_2_) have been reported for tetra‐*bay*‐arylated peropyrenes [18], indicating a twist‐dependent influence on the excited‐state lifetime although possible solvent‐dependent mixing effects with the dark S_2_ state should be borne in mind. Compared to that, the slightly increasing trend in lifetimes for the aza‐derivatives points to a subtle (positional) N‐doping effect, which is further supported by the natural lifetimes (*τ*_0_) of 3.63, 3.83, and 4.17 ns for **4c**, **4a**, and **4b**, respectively. Fluorescence quantum yields (*Φ*_F_) are in the range of 69%–73% (Figure 5b, Table 1) and are higher than those previously reported for all‐carbon analogues [14, 16] with electron rich aryl substituents (*Φ*_F_  =  53%–62% in toluene), while modestly lower than that of the parent planar **PP** [10] (*Φ*_F_  =  86% in toluene). The similarity in *Φ*_F_ to a single *bay*‐phenyl‐substituted PP [19] derivative (*Φ*_F_  =  75%) further indicates that one phenyl group per *bay* region has little impact on the fluorescence quantum yield, unlike the significantly decreased *Φ*_F_ values observed when two aryl groups occupy the same *bay* regions (*Φ*_F_  =  23%–48%) [15, 18]. N‐doping at the 1‐ or 2‐position shows no significant effect on the emission efficiency, as also shown for a twisted diazaperopyrene [31] doped at the 2,9‐positions (*Φ*_F_  =  75%). This contrasts with the relatively low quantum yields reported for twisted 1,3,8,10‐tetrazaperopyrenes (*Φ*_F_  =  5%–12%) [32].

In the solid state, the (A)PP derivatives exhibit pronounced fluorescence modulation, highlighting the markedly different aggregation behavior and excited state deactivation pathways as compared to the solution state (Figure 5f). The as‐prepared solid of all‐carbon peropyrene **4c** displays yellow fluorescence, which changes to yellow‐green upon grinding. The unground solids of the nitrogen‐containing peropyrenes **4a** and **4b** exhibit typical aggregation‐induced quenching (AIQ) behavior and are nonemissive, but become faintly yellow‐orange and red emissive, respectively, upon mechanical stimulation.

### 2.5 Acidochromism

**FIGURE 6:**
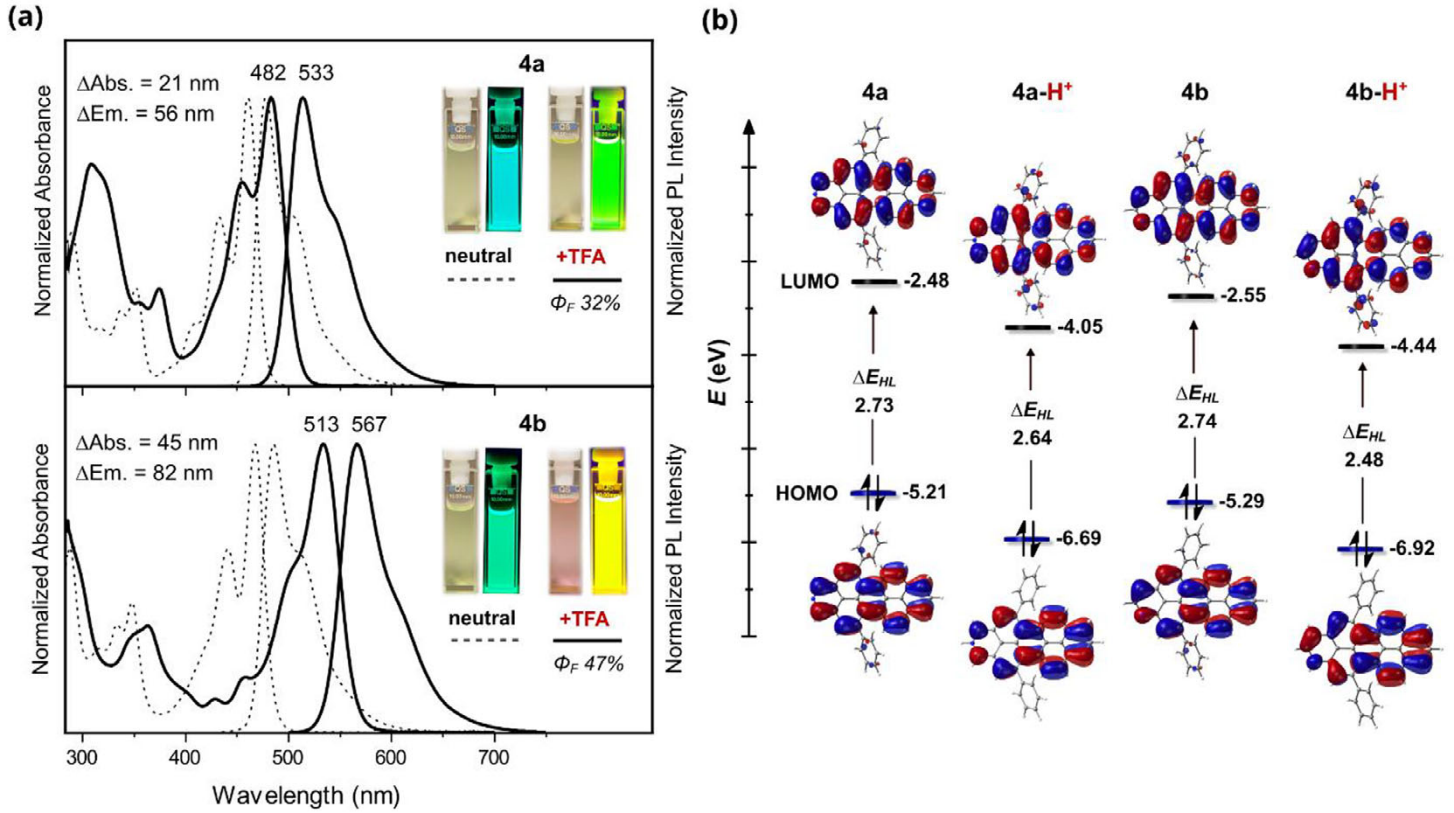
(a) absorption and emission spectra of **4a** and **4b** in toluene before (dashed line) and after (solid line) addition of TFA (c ≈ 1.0 × 10^−5^ M, 20 °C). Insets: photographs of the corresponding solutions before and after addition of TFA taken under daylight (left) and UV‐irradiation (right) (*λ*_ex_  =  365 nm). (b) Frontier molecular orbitals, energy levels and HOMO‐LUMO gaps (Δ*E*_HL_) of neutral and protonated **4a** and **4b** calculated at the B3LYP‐D3(BJ)/6−311G(d,p) level (isovalue  =  0.02).

The presence of the pyridinic nitrogen also prompted us to examine the behavior of **4a** and **4b** as proton acceptors. As shown in Figure 6a, addition of one drop of trifluoroacetic acid (TFA) to diluted toluene solutions of the azaperopyrenes results in proton‐triggered optical switching with redshifts of up to 45 nm in the absorption and 82 nm in the emission. The *p*‐band absorption maximum of **4a** shifts bathochromically by 21 to 482 nm, accompanied by a fluorescence color change from cyan to green with an emission maximum at 533 nm. Protonation of **4b** affords a magenta‐colored solution with a *p*‐band maximum at 513 nm and yellow emission centered at 567 nm. Both protonated **4a** and **4b** retain vibronically structured absorption and emission spectra of near mirror symmetry, with Stokes shifts of 1985 and 1856 cm^−1^, and absolute fluorescence quantum yields of 32% and 47%, respectively. Additional acid causes no further spectral changes, and the original spectra can be restored by adding triethylamine (TEA). As in the neutral species, the *p*‐absorption bands centered at 482 and 513 nm are assigned to the HOMO → LUMO transition (Tables S22,23). The HOMO and LUMO of protonated **4a** and **4b** remain delocalized over the core structure. Protonation leads to significant stabilization of both frontier orbitals, with a more pronounced stabilization of the LUMO in **4b**, resulting in a narrower HOMO‐LUMO gap compared to **4a** (Figure 6b). This acidochromism further illustrates the combined effect of nitrogen incorporation and its position on the photophysics of peropyrene.

### 2.6 Redox Properties and Frontier Orbital Energies

**FIGURE 7:**
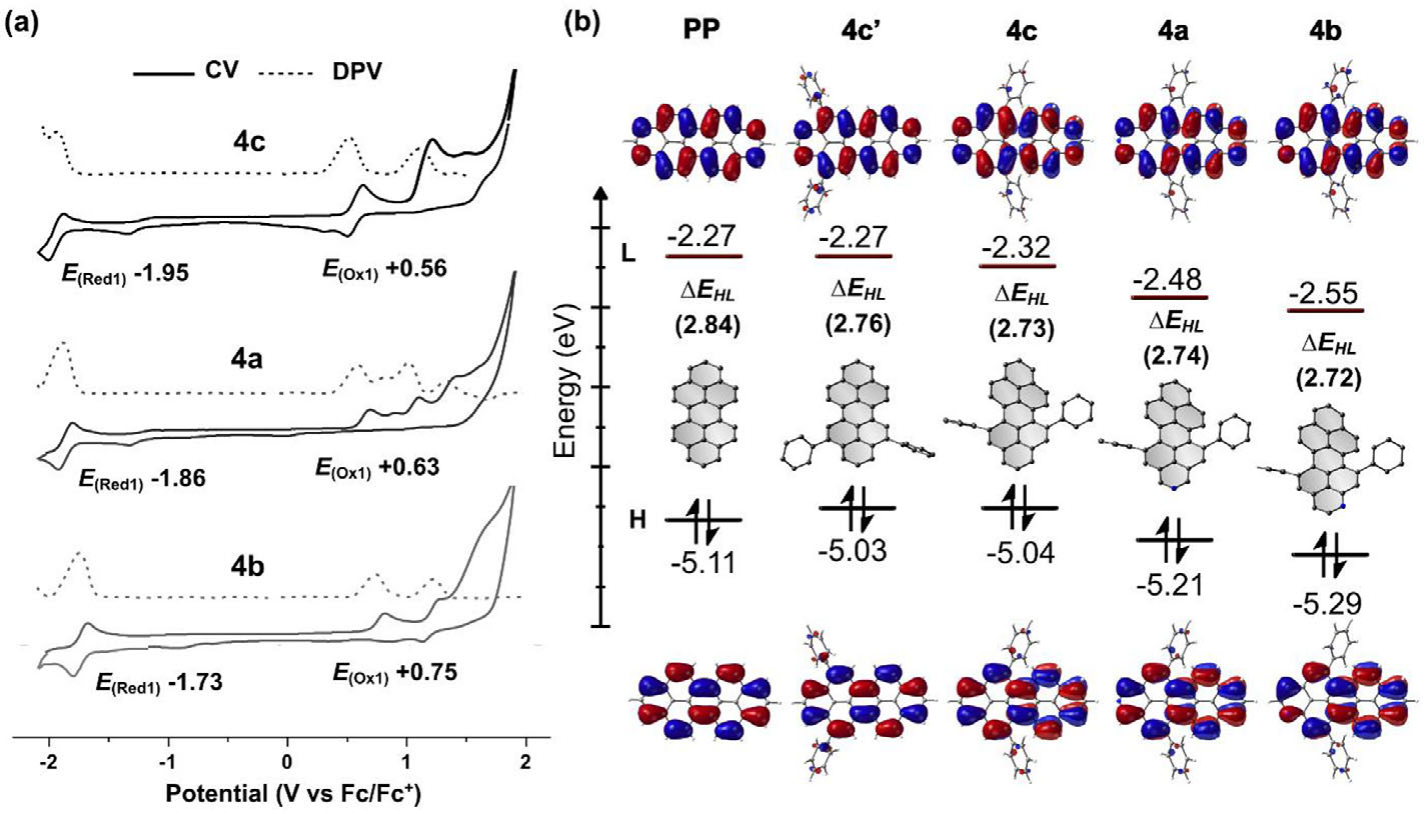
(a) Cyclic voltammetry (CV) and differential pulse voltammetry (DPV) curves in CH_2_Cl_2_ containing 0.1 M supporting electrolyte (Bu_4_NPF_6_). Reference is ferrocene (Fc/Fc^+^), scan rate = 0.1 V/s, *c* ≈ 1.0 × 10^−3^ M, 20 °C. (b) Frontier molecular orbitals and energy levels illustrating the effects of functional‐group substitution position, molecular twisting, and nitrogen doping on the HOMO and LUMO energies and the HOMO‐LUMO gap (Δ*E*_HL_), calculated at the B3LYP‐D3(BJ)/6‐311G(d,p) level of theory.

To evaluate the position‐dependent nitrogen doping effect on the redox properties, cyclic voltammetry (CV) and differential pulse voltammetry (DPV) measurements were performed in CH_2_Cl_2_. The CV and DPV voltammograms are shown in Figure 7a, and the corresponding electrochemical data are summarized in Tables S18. The lowest oxidation potential of all‐carbon peropyrene **4c** is observed at +0.56 V with good reversibility, while a partially reversible reduction occurs at −1.95 V (vs. Fc/Fc⁺), consistent with reported redox values for related 5,13‐*bay*‐arylated PPs [17]. Analogous to the electrochemical behavior observed for the lower 1‐ and 2‐azapyrene homologues [34, 35] and other N‐doped peropyrenes [24, 26, 31], **4a** and **4b** exhibit irreversible oxidation waves. Relative to **4c**, the first oxidation potential is positively shifted to +0.63 V for **4a** and further to +0.75 V for **4b**, while reversible reductions occur at less negative potentials of −1.86 V and −1.73 V (vs. Fc/Fc⁺), respectively. Accordingly, **4a** and **4b** exhibit higher oxidation (Δ*E*_ox_  =  0.07 (**4a**) and 0.19 (**4b**) V) and reduction potentials (Δ*E*_red_  =  0.09 (**4a**) and 0.22 (**4b**) V) compared to **4c**, which correlates well with the observed trends in the HOMO and LUMO energies (Figure 7b). Comparison of the frontier orbital energies of **4c** with those of parent **PP** and the planar positional isomer **4c’** (Figures 7b and S9) further reveals that twisting of the PP backbone predominantly influence the LUMO, while incorporation of nitrogen stabilizes both frontier orbitals, without significantly altering the HOMO‐LUMO gap.

## 3 Summary

In conclusion, we developed two novel approaches for the synthesis of hitherto unknown 1‐ and 2‐azaperopyrene derivatives. The synthetic strategy is based on chemoselective cross‐coupling reactions followed by Brønsted‐acid mediated cyclization and was further applied to the synthesis of an all‐carbon peropyrene derivative, avoiding the requirement of not readily available bis(arylalkynyl)phenylboronates. Crystal structure analysis reveal twisted conformations with *bay* and end‐to‐end twist angles varying between 10.0–19.9° and 5.5–25.0° for the same number and steric demand of *bay* substituents, which was attributed to crystal packing effects. Further analysis of the crystal structures discloses the presence of conformational isomorphism with two symmetry‐independent pairs of enantiomers in the same crystal lattice. Evaluation of multiple aromaticity descriptors indicate that the central ring of peropyrene exhibits reduced aromaticity, while its overall aromatic character is largely unaffected by twisting and N‐doping. All three synthesized peropyrenes show intense absorption in the visible region and high fluorescence quantum yields. The optical properties can be further tuned by protonation of the pyridine moiety. Simultaneously, N‐doping stabilize the HOMO and LUMO energy levels without appreciably altering the band gap. Further studies will be devoted to the synthesis of functionalized (aza)peropyrenes and evaluation of their potential singlet‐fission‐properties.

## Conflicts of Interest

The authors declare no conflict of interest.

## Supporting information




**Supporting File 1**: chem70603‐sup‐0001‐SuppMat.docx

**Supporting File 2**: chem70603‐sup‐0002‐SuppMat.zip

## Data Availability

The data that support the findings of this study are available in the supplementary material of this article.
