# Supplementary material for: Twisted 1‐ and 2‐Azaperopyrenes: Synthesis, Structure, and Properties
Source: Chemistry. 2025 Dec 31;32(8):e03136. doi: 10.1002/chem.202503136 (PMC12929927; doi:10.1002/chem.202503136)
Supplement: Supplementary file 1 — Supporting File 1: chem70603‐sup‐0001‐SuppMat.docx [file CHEM-32-e03136-s001.docx]

*Supporting Information*

Twisted 1- and 2-Azaperopyrenes: Synthesis, Structure and Photophysical Properties

Ricardo Molenda^a^, Arpine Vardanyan^a^, Alexander Villinger^a^, Peter Ehlers^a,b^, Peter Langer^a,b^*

^a^ Universität Rostock, Institut für Chemie, A.-Einstein-Str. 3a, 18059 Rostock, Germany Tel.: +49 381 498 6410, Fax : +49 381 498 6412.

^b^ Leibniz Institut für Katalyse an der Universität Rostock, A.-Einstein-Str. 29a, 18059 Rostock, Germany

*Corresponding author: [peter.langer@uni-rostock.de](mailto:peter.langer@uni-rostock.de); web: [www.langer.chemie.uni-rostock.de](http://www.langer.chemie.uni-rostock.de)

Table of Contents

[**1.** **General Information** S2](#_Toc216228669)

[**2.** **Synthesis** S3](#_Toc216228670)

[2.1. Optimization reactions S3](#_Toc216228671)

[2.2. Synthetic Procedures and Characterization S6](#_Toc216228672)

[**3.** **X-ray crystallographic analysis** S16](#_Toc216228673)

[**4.** **Electronic structure and Aromaticity** S17](#_Toc216228674)

[4.1. Bond length analysis S17](#_Toc216228675)

[4.2. HOMA(c) S19](#_Toc216228676)

[4.3. NICS S20](#_Toc216228677)

[4.4. NICS2BC S21](#_Toc216228678)

[4.5. ACID-π S22](#_Toc216228679)

[4.6. LOL-π maps S22](#_Toc216228680)

[4.7. Multicenter Bond order (MCBO) S24](#_Toc216228681)

[4.8. AV1245 S24](#_Toc216228682)

[**5.** **Photophysical Data** S25](#_Toc216228683)

[**6.** **Electrochemical Data** S26](#_Toc216228684)

[**7.** **Computational Studies** S27](#_Toc216228685)

[**8.** **NMR Spectra** S49](#_Toc216228686)

[**9.** **References** S61](#_Toc216228687)

# **General Information**

All chemicals (reagents, catalysts, ligands, acids, and bases) were obtained from commercial suppliers and used without further purification, unless otherwise stated. Solvents employed in reactions were purchased as extra dry under argon. Solvents used for extraction and column chromatography were distilled prior to use. Thin layer chromatography (TLC) was performed on Supelco® TLC Silica gel 60 F254 on aluminum foil. Column chromatography was carried out on silica gel 60 (0.035–0.070 mm) or silica gel 60 (0.06–0.200 mm).

The nuclear magnetic resonance spectra (^1^H/^13^C/^19^F NMR) were recorded on a Bruker AVANCE 250 II, 300 III, NEO 500 or Jeol ECZL 400. Chemical shifts (δ) are given in parts per million (ppm) and are referenced to the residual deuterated chloroform signal (CDCl₃: δ = 7.26 ppm (^1^H), 77.16 ppm (^13^C)). Multiplicities due to spin–spin correlation are reported as follows: s = singlet, d = doublet, t = triplet, dd = double doublet, m = multiplet; they are further described through their coupling constants *J* expressed in Hz.

Basic and high-resolution mass spectra (MS/HRMS) were measured on instruments, which are paired with a preceding gas chromatograph (GC) or liquid chromatograph (LC). The samples have been ionized through electron impact ionization (EI) on an Agilent 6890/5973 or Agilent 7890/5977 GC–MS equipped with a HP-5 capillary column using helium carrier gas or by applying electron spray ionization (ESI) on an Agilent 1200/6210 Time-of-Flight (TOF) LC–MS. X-ray single-crystal structure analysis was performed on a Bruker Apex Kappa-II CCD diffractometer. Infrared spectra (IR) were measured as attenuated total reflection (ATR) experiments with a Bruker FTIR ALPHA II spectrometer. The signals have been characterized through their wave numbers and their corresponding absorption as very strong (vs), strong (s), medium (m) or weak (w). Melting points (mp) were determined by Micro-Hot-Stage GalenTM III Cambridge Instruments and are not corrected.

UV/Vis spectra were recorded on an Agilent Cary 60 UV–vis spectrophotometer and emission spectra with an Agilent Cary Eclipse fluorescence spectrophotometer. Fluorescence lifetimes were measured on an Edinburgh Instruments FS5 v2 spectrofluorometer using an EPL-405 (λ_ex_ = 404.8 nm) picosecond pulsed diode laser for excitation, with detection via time-correlated single-photon counting (TCSPC). Quartz cuvettes (1 cm) and spectroscopy-grade solvents were used for all measurements. Relative fluorescence quantum yields (Φ*_F_*) were determined using coumarin 153 in ethanol (Φ*_F_* = 0.4) as the standard.^[1]^ Absolute fluorescence quantum yields were measured using an Edinburgh Instruments FS5 v2 spectrofluorometer with an integrated sphere module. The optical density (OD) of the sample at the excitation wavelength was set to OD = 0.1.

Cyclic voltammetry (CV) and differential pulse voltammetry (DPV) measurements were performed under argon at room temperature using a Parstat 4000 potentiostat (Princeton Applied Research) in a three-electrode configuration with anhydrous CH₂Cl₂ as solvent and 0.1 M *n*Bu₄NPF₆ as supporting electrolyte. A glassy carbon disk electrode (3 mm diameter, KeI-F coating, length 80 mm, 6.35 mm OD), polished with 0.03 μm alumina slurry, was used as working electrode. The reference electrode was Ag/AgNO₃ (0.01 M in CH₃CN; ANE2), and the counter electrode was a platinum wire (0.5 mm diameter, 6.35 mm OD). Measurements were carried out at a scan rate of 100 mV s⁻¹ with sample concentrations of ca. 1 mM. Ferrocene (1 mM in CH₃CN) was used as external standard, and all potentials are reported vs Fc/Fc^+^. Voltammograms were plotted according to the IUPAC convention.

# **Synthesis**

## Optimization reactions

- - 1. Optimization of **3a** (route A)

**Table S1**. Optimization of **3a** (route A).

| entry | catalyst/ligand ((5/10) mol%) | 2-(Bpin)pyrene eq. | base  (2 eq.) | solvent | temp. (°C) | time  (h) | yield (%) ^a^ |
| --- | --- | --- | --- | --- | --- | --- | --- |
| 1 | **Pd(PPh_3_)_4_** | **1.2** | **K_3_PO_4_** | **1,4-dioxane/H_2_O** | **100** | **7** | **63** |
| 2 | Pd(PPh_3_)_4_ | 1.5 | K_3_PO_4_ | 1,4-dioxane/H_2_O | 100 | 5 | 64 |
| 3 | Pd(PPh_3_)_4_ | 1.2 | K_3_PO_4_ | 1,4-dioxane/H_2_O | 100 | 24 | 63 |
| 4 | Pd(PPh_3_)_4_ | 1.2 | K_3_PO_4_ | Toluene | 100 | 24 | traces^b^ |
| 6 | Pd(PPh_3_)_4_ | 1.2 | K_2_CO_3_ | toluene/EtOH/H_2_O | 100 | 24 | 60 |
| 7 | Pd(PPh_3_)_4_ | 1.2 | K_2_CO_3_ | THF/H_2_O | 80 | 24 | 58 |
| 5 | Pd(CH_3_CN)_2_Cl_2_/  SPhos | 1.2 | K_3_PO_4_ | toluene | 100 | 24 | traces^b^ |

^a^ Yield after column chromatography and subsequent washing with methanol. ^b^ Not determined.

- - 1. Optimization of **3b** (route A)

**Table S2**. Optimization of **3b** (route A).^a^

| entry | catalyst  (mol %) | ligand  (mol %) | base | solvent | temp (°C) | time (h) | yield  (%) |
| --- | --- | --- | --- | --- | --- | --- | --- |
| 1 | Pd_2_dba_3_ (5) | SPhos (10) | K_3_PO_4_ | toluene | 110 | 24 | - |
| 2 | Pd(PPh_3_)_4_ (5) | - | K_2_CO_3_ | 1,4-dioxane/H_2_O | 90 | 24 | 20 |
| 3 | Pd(PPh_3_)_4_ (5) | SPhos (10) | K_2_CO_3_ | 1,4-dioxane/H_2_O | 90 | 24 | 8 |
| 4 | Pd(PPh_3_)_4_ (5) | XPhos (10) | K_2_CO_3_ | 1,4-dioxane/H_2_O | 100 | 24 | 23 |
| **5** | **PdCl_2_(MeCN)_2_ (5)** | **XPhos (10)** | **K_3_PO_4_** | **1,4-dioxane/H_2_O** | **100** | **24** | **69** |
| 6 | Pd(OAc)_2_ (7.5) | DPEphos (8) | K_3_PO_4_ | toluene | 100 | 24 | traces |

^a^ All reactions were performed with 2 eq. 2-(Bpin)pyrene.

- - 1. Optimization of **3a** (route B)

**Table S3**. Optimization of **3a** (route B).

| entry | catalyst  (5-mol%) | ligand (10-mol%) | co-catalyst ^a^ | base | alkyne  (eq.) | solvent | temp. (°C) | time (h) | yield (%) |
| --- | --- | --- | --- | --- | --- | --- | --- | --- | --- |
| 1 | PdCl_2_(MeCN)_2_ | XPhos | CuI | DIPA | 3 | 1,4-dioxane | 100 | 24 | 0 |
| 2 | PdCl_2_(MeCN)_2_ | XPhos | CuI | DIPA | 5 | 1,4-dioxane | 100 | 24 | 0 |
| 3 | PdCl_2_(MeCN)_2_ | P*t*Bu_3_-HBF_4_ | CuI | DIPA | 3 | 1,4-dioxane | 100 | 24 | 0 |
| 4 | PdCl_2_(MeCN)_2_ | CataCXium A | CuI | DIPA | 3 | MeCN | 80 | 24 | 0 |
| 5 | Pd(PPh_3_)_2_Cl_2_ | CataCXium A | CuI | DIPA | 3 | MeCN | 80 | 24 | 0 |
| 6 | Pd(OAc)_2_ | XPhos | CuI | DIPA | 3 | MeCN | 80 | 24 | 0 |
| 7 | Pd(OAc)_2_ | P*t*Bu_3_-HBF_4_ | CuI | DIPA | 3 | MeCN | 80 | 24 | 0 |
| 8 | Pd(PPh_3_)_4_ | - | CuI | DIPA | 3 | MeCN | 80 | 24 | 0 |
| 9 | Pd(PPh_3_)_2_Cl_2_ | - | CuI | DIPA | 3 | DMF | 110 | 24 | 0 |
| 10 | Pd(dppf)Cl_2_ | - | CuI | DIPA | 3 | THF | 80 | 24 | 0 |
| 11 ^b^ | **Pd/C 10 wt.%** | **XPhos** | **-** | **K_2_CO_3_** | **3** | **DMF** | **110** | **2** | **86** |

^a^ Reactions were performed with 5-mol% CuI. ^b^ 7-mol% Pd. DIPA = diisopropylamine.

- - 1. Optimization of **4a**

**Table S4**. Optimization of **4a**.

| entry | Brønsted acid | eq. | solvent | temp. (°C) | time (h) | yield (%) |
| --- | --- | --- | --- | --- | --- | --- |
| 1 | MsOH | 10 | - | 120 | 24 | nd **^a^** |
| 2 | MsOH | 15 | - | 120 | 6 | 29 |
| 3 | MsOH | 15 | - | 100 | 11 | 67 |
| 4 | MsOH | 30 | - | 100 | 9 | 43 |
| 5 | *p*TsOH⋅H_2_O | 15 | - | 100 | 24 | nd **^a^** |
| 6 | ***p*TsOH⋅H_2_O** | **20** | **-** | **100** | **12** | **79** |
| 7^b^ | TfOH | 5 | CH_2_Cl_2_ | 0-rt | 3 | 64 |

nd: Not determined. **^a^** Incomplete reaction. ^b^ Inert atmosphere.

## Synthetic Procedures and Characterization

4-chloro-3,5-bis(phenylethynyl)pyridine ^[2]^ (**2a**), 3-chloro-2,4-bis(phenylethynyl)pyridine (**2b**) ^[3]^, 3,5-dichloro-4-iodopyridine (**1c**) ^[4]^, 1,3-dibromo-2-iodobenzene (**1f**) ^[5]^ and 1,3-dichloro-2-iodobenzene (**1e**) ^[6]^ were prepared according to literature procedures.

- - 1. **Synthesis of 2-azaperopyrene 4a**

**3,5-bis(phenylethynyl)-4-(pyren-2-yl)pyridine (3a)**

**Route A**:

A pressure tube charged with **2a** (200 mg, 0.637 mmol, 1 eq.), 2-(Bpin)pyrene (250.9 mg, 0.764 mmol, 1.2 eq.), K_3_PO_4_ (270.4 mg, 1.274 mmol, 2 eq.) and Pd(PPh_3_)_4_ (36.8 mg, 31.9 µmol, 0.05 eq.) was evacuated and backfilled with argon for 3 times. Then 1,4-dioxane (3 ml) and distilled water (1 ml) were added. The pressure tube was sealed with a teflon cap and the reaction mixture was stirred at 100 °C for 7h h in a stainless steel heating block. After cooling to room temperature, the reaction was quenched with distilled water and extracted with EtOAc (3x). The combined organic layers were dried over Na_2_SO_4_, filtered, concentrated in vacuo and roughly purified by silica gel column chromatography (EtOAc *v*/*v* = 10:1). Final purification was achieved by repeated washing of the pre-purified residue with small amounts of MeOH, until TLC showed no remaining impurities. The title compound was isolated as an off-white solid in 63% yield (192.7 mg), heptane/EtOAc *v*/*v* = 10:1; **mp** 212 °C. **^1^H NMR** (500 MHz, CDCl_3_): *δ* = 7.13 – 7.17 (m, 8H), 7.18 – 7.23 (m, 2H), 8.07 (t, *J* = 7.6 Hz, 1H), 8.13 (d, *J* = 8.9 Hz, 2H), 8.16 (d, *J* = 8.9 Hz, 2H), 8.24 (d, *J* = 7.6 Hz, 2H), 8.60 (s, 2H), 8.91 (s, 2H). **^13^C{^1^H} NMR** (126 MHz, CDCl_3_): *δ* = 85.8, 95.6, 119.8, 122.3, 124.56, 124.65, 125.2, 126.3, 126.5, 127.4, 127.8, 128.2, 128.6, 130.6, 131.4, 133.5, 151.6, 151.9. One quaternary carbon signal is not visible in the ^13^C{^1^H} NMR spectrum. **IR** (ATR, cm^-1^): ṽ = 686 (vs), 748 (s), 886 (s), 1488 (m), 2211 (w), 3033 (w). **MS** (EI, 70 eV): *m/z* (%) = 479 (100, M^+^), 478 (89), 477 (37), 476 (27), 475 (26), 402 (13), 401 (19), 239 (19), 238 (26), 225 (10), 224 (13). **HRMS** (EI): calcd for C_37_H_21_N [M^+^] 479.1668, found 479.1666.

**Route B**:

A pressure tube charged with 3,5-dichloro-4-(pyren-2-yl)pyridine **2c** (100 mg, 0.287 mmol, 1 eq.), XPhos (8.20 mg, 17.2 µmol, 0.06 eq.), K_2_CO_3_ (119.0 mg, 0.861 mmol, 3 eq.) and Pd/C (10 wt%) (21.4 mg, 20.0 µmol Pd, 0.07 eq.) was evacuated and backfilled with argon for 3 times. Then dry DMF (4 ml) was added followed by drop wise addition of phenylacetylene (87.9 mg, 94.6 µl, 0.861 mmol, 3 eq.). The pressure tube was sealed with a teflon cap and the reaction mixture was stirred at 110 °C for 2 h in a stainless steel heating block. After cooling to room temperature, the reaction was quenched with distilled water and extracted with CH_2_Cl_2_ (3x). The combined organic layers were dried over Na_2_SO_4_, filtered, concentrated in vacuo and purified by silica gel column chromatography to obtain **3a** in 86% yield (118.6 mg), heptane/EtOAc *v*/*v* = 4:1.

**3,5-dichloro-4-(pyren-2-yl)pyridine (2c)**

A pressure tube charged with 3,5-dichloro-4-iodopyridine **1c** (330.0 mg, 1.205 mmol, 1 eq.), 2-(BPin)pyrene (474.6 mg, 1.446 mmol, 1.2 eq.), K_3_PO_4_ (511.6 mg, 2.410 mmol, 2 eq.) and Pd(PPh_3_)_4_ (69.6 mg, 60.2 µmol 0.05 eq.) was evacuated and backfilled with argon for three times. Then 1,4-dioxane (10 ml) and distilled water (1.7 ml) were added. The pressure tube was sealed with a teflon cap and stirred at 90 °C for 48 h in a stainless steel heating block. After cooling to room temperature, the reaction was quenched with distilled water and extracted with EtOAc (3x). The combined organic layers were dried over Na_2_SO_4_, filtered, concentrated in vacuo and purified by silica gel column chromatography to obtain **2c** as a colorless solid in 76% yield (317.8 mg), heptane/EtOAc *v*/*v* = 15:1; **mp** 193 – 195 °C. **^1^H NMR** (500 MHz, CDCl_3_): *δ* = 8.04 – 8.08 (m, 1H), 8.08 (s, 2H), 8.11 (d, *J* = 8.9 Hz, 2H), 8.15 (d, *J* = 8.9 Hz, 2H), 8.24 (d, *J* = 7.6 Hz, 2H), 8.69 (s, 2H). **^13^C{^1^H} NMR** (126 MHz, CDCl_3_): *δ* = 124.4, 124.6, 124.9, 125.5, 126.5, 127.2, 128.2, 131.2, 131.3, 131.4, 132.4, 147.1, 147.8. **IR** (ATR, cm^-1^): ṽ = 711 (vs), 820 (s), 841 (s), 882 (m), 1210 (s), 1391 (m), 1562 (w), 3035 (w). **MS** (EI, 70 eV): *m/z* (%) = 347 (100, M^+^), 277 (26), 275 (14), 250 (15), 248 (11), 125 (22), 124 (16). **HRMS** (ESI): calcd for C_21_H_11_Cl_2_N [M+H]^+^ 348.0347, found 348.0347.

**5,13-diphenylbenzo[12,1]tetrapheno[9,8,7-*def*]isoquinoline (4a)**

A pressure tube charged with **3a** (100 mg, 0.209 mmol, 1 eq.) and *p*TsOH⋅H_2_O (793.2 mg, 4.17 mmol, 20 eq.) was sealed with a teflon cap and stirred at 100 °C for 12 h in a stainless steel heating block. After cooling to room temperature, the reaction mixture was neutralized with a saturated aqueous solution of NaHCO_3_ and extracted with CH_2_Cl_2_ (3x). The combined organic layers were dried over Na_2_SO_4_, filtered, concentrated in vacuo and purified by silica gel column chromatography to obtain **4a** as an orange solid in 79% yield (79.3 mg), heptane/EtOAc *v*/*v* = 3:1; **mp** 344 – 345 °C. **^1^H NMR** (500 MHz, CDCl_3_): *δ* = 7.49 – 7.55 (m, 10H), 7.80 (d, *J* = 9.4 Hz, 2H), 8.05 – 8.09 (m, 1H), 8.18 – 8.22 (m, 4H), 8.26 (s, 2H), 9.63 (s, 2H). **^13^C{^1^H} NMR** (75 MHz, CDCl_3_): *δ* = 123.98, 123.99, 125.2, 125.3, 125.5, 125.7, 126.5, 126.6, 127.4, 127.9, 128.2, 129.0, 129.1, 130.8, 141.3, 145.2. Two quaternary and one CH carbon signals are not visible in the ^13^C{^1^H} NMR spectrum. **IR** (ATR, cm^-1^): ṽ = 699 (vs), 760 (vs), 808 (s), 839 (s), 896 (s), 1152 (m), 1442 (m), 1488 (m), 2850 (m), 2920 (m). **MS** (EI, 70 eV): *m/z* (%) = 479 (100, M^+^), 478 (34), 477 (18), 476 (19), 475 (21), 239 (17), 238 (36), 231 (17), 224 (12). **HRMS** (ESI): calcd for C_37_H_21_N [M+H]^+^ 480.1752, found 480.1762.

- - 1. **Synthesis of 1-azaperopyrene 4b**

**2,4-bis(phenylethynyl)-3-(pyren-2-yl)pyridine (3b)**

**Route A:**

A pressure tube charged with **2b** (100.0 mg, 0.319 mmol, 1 eq.), 2-(Bpin)pyrene (209.4 mg, 0.638 mmol, 2 eq.), K_3_PO_4_ (203.1 mg, 0.957 mmol, 3 eq.), XPhos (15.2 mg, 31.9 µmol, 0.1 eq.) and PdCl_2_(MeCN)_2_ (4.15 mg, 16.0 µmol, 0.05 eq.) was evacuated and backfilled with argon for 3 times. Then 1,4-dioxane (3 ml) and distilled water (1 ml) were added and the pressure tube was sealed with a teflon cap. The reaction mixture was stirred at 100 °C for 24 h in a stainless steel heating block. After cooling to room temperature, the reaction was quenched with distilled water and extracted with EtOAc (3x). The combined organic layers were dried over Na_2_SO_4_, filtered, concentrated in vacuo and purified by silica gel column chromatography to obtain **3b** as a yellow solid in 69% yield (106.2 mg), heptane/EtOAc *v*/*v* = 2:1; **mp** 192 °C. **^1^H NMR** (300 MHz, CDCl_3_): *δ* = 7.03 – 7.24 (m, 10H), 7.56 (d, *J* = 5.0 Hz, 1H), 8.07 (dd, *J* = 8.2 Hz, *J* = 7.1 Hz, 1H), 8.13 (d, *J* = 9.0 Hz, 2H), 8.17 (d, *J* = 9.0 Hz, 2H), 8.25 (d, *J* = 7.6 Hz, 2H), 8.55 (s, 2H), 8.68 (d, *J* = 5.1 Hz, 1H). **^13^C{^1^H} NMR** (63 MHz, CDCl_3_): *δ* = 86.6, 88.7, 92.8, 97.5, 121.8, 122.0, 124.4, 124.6, 125.1, 125.2, 126.2, 127.0, 127.5, 127.7, 128.1, 128.3, 128.8, 129.1, 130.6, 131.4, 131.7, 131.8, 134.1, 140.9, 143.0, 148.7. One quaternary carbon signal is not visible in the ^13^C{^1^H} NMR spectrum. **IR** (ATR, cm^-1^): ṽ = 686 (vs), 710 (s), 752 (vs), 816 (s), 838 (s), 886 (m), 1392 (m), 1489 (m), 1555 (m), 2209 (w). **MS** (EI, 70 eV): *m/z* (%) = 479 (100, M^+^), 478 (95), 476 (31), 239 (36), 238 (51), 237 (22). **HRMS** (ESI): calcd for C_37_H_22_N [M+H]^+^ 480.1752, found 480.1753.

**Route B:**

A pressure tube charged with 2,4-dichloro-3-(pyren-2-yl)pyridine **2d** (100.0 mg, 0.287 mmol, 1 eq.), XPhos (8.20 mg, 17.2 µmol, 0.06 eq.), K_2_CO_3_ (119.0 mg, 0.861 mmol, 3 eq.) and Pd/C (10 wt%) (21.4 mg, 20.0 µmol Pd, 0.07 eq.) was evacuated and backfilled with argon for 3 times. Then dry DMF (4 ml) was added followed by drop wise addition of phenylacetylene (87.9 mg, 94.6 µl, 0.861 mmol, 3 eq.). The pressure tube was sealed with a teflon cap and the reaction mixture was stirred at 110 °C for 4 h in a stainless steel heating block. After cooling to room temperature, the reaction was quenched with distilled water and extracted with EtOAc (3x). The combined organic layers were dried over Na_2_SO_4_, filtered, concentrated in vacuo and purified by silica gel column chromatography (heptane/EtOAc *v*/*v* = 2:1) to obtain **3b** as an off-white solid in 66% yield (90.6 mg).

**2,4-dichloro-3-(pyren-2-yl)pyridine (2d)**

A pressure tube charged with 2,4-dichloro-3-iodopyridine **1d** (100 mg, 0.365 mmol, 1 eq.), 2-(Bpin)pyrene (143.8 mg, 0.438 mmol, 1.2 eq.), K_3_PO_4_ (233.5 mg, 1.1 mmol, 3 eq.) and Pd(PPh_3_)_4_ (21.1 mg, 18.3 µmol, 0.05 eq.) was evacuated and backfilled with argon for 3 times. Then 1,4-dioxane (3 ml) and distilled water (0.5 ml) were added and the pressure tube was sealed with a teflon cap. The reaction mixture was stirred at 90 °C for 24 h in a stainless steel heating block. After cooling to room temperature, the reaction was quenched with distilled water and extracted with EtOAc (3x). The combined organic layers were dried over Na_2_SO_4_, filtered, concentrated in vacuo and purified by silica gel column chromatography (heptane/EtOAc *v*/*v* = 10:1 ) to obtain **2d** as an off-white solid in 79% yield (100.6 mg); **mp** 171 °C. **^1^H NMR** (500 MHz, CDCl_3_): *δ* = 7.49 (d, *J* = 5.3 Hz, 1H), 8.05 (t, *J* = 7.6 Hz, 1H), 8.09 – 8.12 (m, 4H), 8.14 (d, *J* = 9.0 Hz, 2H), 8.23 (d, *J* = 7.6 Hz, 2H), 8.39 (d, *J* = 5.3 Hz, 1H). **^13^C{^1^H} NMR** (126 MHz, CDCl_3_): *δ* = 124.0, 124.4, 124.5, 125.4, 125.5, 126.3, 127.2, 128.1, 131.3, 132.3, 136.3, 145.8, 148.6, 152.2. One quaternary carbon signal is not visible in the ^13^C{^1^H} NMR spectrum. **IR** (ATR, cm^-1^): ṽ = 711 (vs), 756 (s), 793 (s), 816 (vs), 1201 (m), 1368 (m), 1428 (m), 1541 (m). **MS** (EI, 70 eV): *m/z* (%) = 349 (64, M^+^), 348 (21), 347 (100, M^+^), 277 (34), 275 (17), 250 (11), 125 (24), 124 (17). **HRMS** (EI): calcd for C_21_H_11_NCl_2_ [M^+^] 347.0263, found 347.0264; calcd for C_21_H_11_NCl^37^Cl [M^+^] 349.0234, found 349.0234.

**5,13-diphenylbenzo[12,1]tetrapheno[9,8,7-*def*]quinoline (4b)**

A schlenk tube charged with **3b** (100 mg, 0.209 mmol, 1 eq.) was evacuated and backfilled with argon for 3 times. Then, dry dichloromethane (3 ml) was added and the reaction mixture was cooled to 0 °C. TfOH (156.5 mg, 92.0 µl, 1.043 mmol, 5 eq.) was added dropwise and the resulting mixture was stirred at 0 °C for 1 h, followed by 2 h at room temperature. After completion of the reaction (as indicated by TLC analysis), the reaction mixture was neutralized with a saturated aqueous solution of NaHCO_3_ and extracted with CH_2_Cl_2_ (3x). The combined organic layers were dried over Na_2_SO_4_, filtered, concentrated in vacuo and purified by silica gel column chromatography to obtain **4b** as an orange-red solid in 78% yield (78.6 mg), heptane/EtOAc *v*/*v* = 2:1; **mp** 331 °C. **^1^H NMR** (300 MHz, CDCl_3_): *δ* = 7.48 – 7.59 (m, 10H), 7.78 – 7.84 (m, 2H), 8.05 (d, *J* = 5.2 Hz, 1H), 8.08 (d, *J* = 7.4 Hz, 1H), 8.15 (s, 1H), 8.18 – 8.24 (m, 4H), 8.48 (s, 1H), 9.32 (d, *J* = 5.1 Hz, 1H). **^13^C{^1^H} NMR** (75 MHz, CDCl_3_): *δ* = 117.4, 118.8, 123.3, 123.8, 124.1, 124.5, 124.6, 125.67, 125.73, 126.1, 126.2, 126.4, 127.5, 127.6, 127.8, 127.9, 128.0, 128.6, 129.06, 129.10, 129.14, 129.2, 130.6, 131.5, 135.7, 143.7, 144.2, 144.9, 145.0, 147.4, 148.5. Two quaternary carbon signals are not visible in the ^13^C{^1^H} NMR spectrum. **IR** (ATR, cm^-1^): ṽ = 682 (s), 700 (vs), 764 (s), 803 (s), 840 (vs), 888 (s), 1026 (m), 1603 (m). **MS** (EI, 70 eV): *m/z* (%) = 479 (100, M^+^), 478 (30), 477 (11), 476 (19), 238 (25), 237 (10), 231 (11). **HRMS** (ESI): calcd for C_37_H_22_N [M+H]^+^ 480.1752, found 480.1762.

- - 1. **Synthesis of peropyrene 4c**

**2-(2,6-dichlorophenyl)pyrene (2e)**

A pressure tube charged with 1,3-dichloro-2-iodobenzene **1e** (200 mg, 0.733 mmol, 1 eq.), 2-(Bpin)pyrene (288.7 mg, 0.880 mmol, 1.2 eq.), K_2_CO_3_ (202.6 mg, 1.466 mmol, 2 eq.) and Pd(PPh_3_)_4_ (42.3 mg, 36.6 µmol, 0.05 eq.) was evacuated and backfilled with argon for 3 times. Then THF (10 ml) and distilled water (2 ml) were added. The pressure tube was sealed with a teflon cap and the reaction mixture was stirred at 80 °C for 48 h in a stainless steel heating block. After cooling to room temperature, the reaction was quenched with distilled water and extracted with CH_2_Cl_2_ (3x). The combined organic layers were dried over Na_2_SO_4_, filtered, concentrated in vacuo and purified by silica gel column chromatography to obtain **2e** as a colorless solid in 71% yield (182 mg), heptane/CH_2_Cl_2_ *v*/*v* = 30:1; **mp** 130 – 131 °C. **^1^H NMR** (400 MHz, CDCl_3_): *δ* = 7.29 – 7.34 (m, 1H), 7.49 – 7.53 (m, 2H), 8.01 – 8.06 (m, 1H), 8.10 – 8.15 (m, 6H), 8.22 (d, *J* = 7.7 Hz, 2H). **^13^C{^1^H} NMR** (101 MHz, CDCl_3_): *δ* = 124.4, 124.7, 125.3, 126.1, 126.2, 127.6, 127.9, 128.3, 129.4, 131.3, 131.4, 134.7, 135.5, 139.9. **IR** (ATR, cm^-1^): ṽ = 711 (vs), 754 (s), 779 (vs), 816 (s), 841 (s), 878 (s), 1084 (m), 1144 (w), 1150 (w), 1177 (m), 1193 (m), 1306 (w), 1426 (s), 1556 (m), 1599 (m), 2852 (m), 2922 (m), 3039 (w). **MS** (EI, 70 eV): *m/z* (%) = 346 (100, M^+^), 277 13, 276 (57), 275 (12), 274 (29), 155 (11), 138 (35), 137 (27). **HRMS** (EI): calcd for C_22_H_12_Cl_2_ [M^+^] 346.0311, found 346.0311; calcd for C_22_H_10_Cl^37^Cl [M^+^] 348.0281, found 348.0286.

**2-(2,6-dibromophenyl)pyrene (2f)**

A pressure tube charged with 1,3-dibromo-2-iodobenzene **1f** (130 mg, 0.359 mmol, 1 eq.), 2-(Bpin)pyrene (141.4 mg, 0.431 mmol, 1.2 eq.), K_2_CO_3_ (99.2 mg, 0.718 mmol, 2 eq.) and Pd(PPh_3_)_4_ (10.4 mg, 9.0 µmol, 0.025 eq.) was evacuated and backfilled with argon for 3 times. Then THF (15 ml) and distilled water (3 ml) were added. The pressure tube was sealed with a teflon cap and the reaction mixture was stirred at 80 °C for 24 h in a stainless steel heating block. After cooling to room temperature, the reaction was quenched with distilled water and extracted with CH_2_Cl_2_ (3x). The combined organic layers were dried over Na_2_SO_4_, filtered, concentrated in vacuo and purified by silica gel column chromatography to obtain **2f** as a colorless solid in 67% yield (105.0 mg), heptane/CH_2_Cl_2_ *v*/*v* = 10:1; **mp** 146 – 147 °C. **^1^H NMR** (300 MHz, CDCl_3_): *δ* = 7.16 (t, *J* = 8.0 Hz, 1H), 7.73 (d, *J* = 8.0 Hz, 2H), 8.02 – 8.06 (m, 3H), 8.10 (d, *J* = 9.0 Hz, 2H), 8.14 (d, *J* = 9.1 Hz, 2H), 8.22 (d, *J* = 7.6 Hz, 2H). **^13^C{^1^H} NMR** (75 MHz, CDCl_3_): *δ* = 124.2, 124.6, 124.9, 125.2, 125.6, 126.1, 127.5, 127.8, 130.0, 131.1, 131.3, 131.9, 138.7, 143.3. **IR** (ATR, cm^-1^): ṽ = 709 (vs), 762 (s), 816 (s), 841 (s), 874 (m), 1140 (m), 1177 (m), 1420 (s), 1545 (m), 1601 (w), 2918 (w), 3035 (w). **MS** (EI, 70 eV): *m/z* (%) = 438 (18, M^+^), 436 (40, M^+^), 434 (17, M^+^), 276 (55), 138 (100), 137 (30), 97 (38), 85 (47), 84 (54). **HRMS** (EI) m/z: [M^+^] Calcd for C_22_H_12_Br_1_^81^Br_1_ 435.9280; Found 435.9278.

**General procedure (A) for the synthesis of 2-(2,6-bis(phenylethynyl)phenyl)pyrene (3c)**

A pressure tube charged with **2e** (100 mg, 0.288 mmol, 1 eq.) or **2f** (100 mg, 0.229 mmol, 1 eq.), K_2_CO_3_ (3 eq.), XPhos (0.06 eq.) and Pd/C (10 wt.%, 0.07 eq. Pd) was evacuated and backfilled with argon for 3 times. Then dry DMF (4 ml) was added followed by dropwise addition of phenylacetylene (3 eq.). The pressure tube was sealed with a teflon cap and the reaction mixture was stirred at 110 °C for 2 h (**2e**) or 24 h (**2f**) in a stainless steel heating block. After cooling to room temperature, the reaction was quenched with distilled water and extracted with CH_2_Cl_2_ (3x). The combined organic layers were dried over Na_2_SO_4_, filtered, concentrated in vacuo and purified by silica gel column chromatography (heptane/CH_2_Cl_2_).

**2-(2,6-bis(phenylethynyl)phenyl)pyrene (3c)**

Following general procedure A, title compound **3c** was isolated in 87% yield (120.4 mg) from **2e** and in 45% yield (49.0 mg) from **2f**.

A pressure tube charged with **2f** (100 mg, 0.229 mmol, 1 eq.), CuI (2.2 mg, 11.4 µmol, 0.05 eq.), XPhos (10.9 mg, 23 µmol, 0.1 eq.) and Pd(OAc)_2_ (2.6 mg, 11.4 µmol, 0.05 eq.) was evacuated and backfilled with argon for 3 times. Then, diisopropylamine (1 ml) and toluene (2 ml) were added followed by dropwise addition of phenylacetylen (70.2 mg, 75.5 µl, 68.7 µmol, 3 eq.). The pressure tube was sealed with a teflon cap and the reaction mixture was stirred at 100 °C for 1 h in a stainless steel heating block. After cooling to room temperature, the reaction mixture was quenched with distilled water and extracted with CH_2_Cl_2_ (3x). The combined organic layers were dried over Na_2_SO_4_, filtered, concentrated in vacuo and purified by silica gel column chromatography to obtain **3c** as a pale yellow solid in 86% yield (94.1 mg), heptane/CH_2_Cl_2_ *v*/*v* = 10:1; **mp**  226 °C. **^1^H NMR** (500 MHz, CDCl_3_): *δ* = 7.01 – 7.05 (m, 4H), 7.06 – 7.10 (m, 4H,), 7.12 – 7.17 (m, 2H), 7.43 (t, *J* = 7.8 Hz, 1H), 7.74 (d, *J* = 7.8 Hz, 2H), 8.05 (t, *J* = 7.6 Hz, 1H), 8.12 (d, *J* = 8.9 Hz, 2H), 8.17 (d, *J* = 8.9 Hz, 2H), 8.57 (s, 2H), 8.23 (d, *J* = 7.6 Hz, 2H). **^13^C{^1^H} NMR** (126 MHz, CDCl_3_): *δ* = 89.0, 93.0, 122.9, 123.7, 124.1, 124.8, 124.9, 125.9, 127.4, 127.7, 128.1, 130.4, 131.3, 131.4, 132.5, 136.4, 145.9. Three CH carbon signals are not visible in the ^13^C{^1^H} NMR spectrum. **IR** (ATR, cm^-1^): ṽ = 690 (vs), 750 (vs), 814 (m), 884 (s), 1177 (m), 1490 (m), 1597 (w), 2850 (m), 2920 (m). **MS** (EI, 70 eV): *m/z* (%) = 478 (23, M^+^), 281 (14), 207 (70), 169 (16), 151 (32), 83 (32), 69 (63), 57 (58). **HRMS** (EI) m/z: [M^+^] Calcd for C_38_H_22_ 478.1716; Found 478.1703.

**5,13-diphenyldibenzo[*cd*,*lm*]perylene (4c)**

An oven-dried, argon flushed Schlenk tube charged with **3c** (100 mg, 0.209 mmol, 1 eq.) and InCl_3_ (46.2 mg, 0.209 mmol, 1 eq.) was evacuated and backfilled with argon for 3 times. Then dry toluene (10 ml) was added and the reaction mixture was stirred at 100 °C for 12 h in a stainless steel heating block. After cooling to room temperature, the reaction was quenched with distilled water and extracted with CH_2_Cl_2_ (3x). The combined organic layers were dried over Na_2_SO_4_, filtered, concentrated in vacuo and purified by silica gel column chromatography to obtain **4c** as a yellow solid in 81% yield (81.1 mg), heptane/CH_2_Cl_2_ *v*/*v* = 10:1; **mp** 331 °C. **^1^H NMR** (500 MHz, CDCl_3_): *δ* = 7.47 – 7.53 (m, 6H), 7.54 – 7.58 (m, 4H), 7.76 (d, *J* = 9.4 Hz, 2H), 8.01 – 8.05 (m, 1H), 8.12 (t, *J* = 7.5 Hz, 1H), 8.17 (d, *J* = 7.5 Hz, 2H), 8.22 (d, *J* = 9.4 Hz, 2H), 8.25 (s, 2H), 8.32 (d, *J* = 7.5 Hz, 2H). **^13^C{^1^H} NMR** (126 MHz, CDCl_3_): *δ* = 123.4, 124.2, 124.39, 124.44, 124.6, 125.2, 125.6, 125.9, 126.1, 126.6, 127.0, 128.3, 129.0, 129.1, 130.8, 130.9, 131.0, 139.6, 145.8. One quaternary carbon signal is not visible in the ^13^C{^1^H} NMR spectrum. **IR** (ATR, cm^-1^): ṽ = 696 (vs), 777 (vs), 839 (s), 890 (s), 1440 (m), 1735 (w), 2852 (m), 2920 (m). **MS** (EI, 70 eV): *m/z* (%) = 478 (100, M^+^), 477 (70), 476 (74), 475 (40), 474 (69), 400 (68), 237 (65), 97 (82), 57 (89). **HRMS** (EI): calcd for C_38_H_22_ [M^+^] 478.1716, found 478.1714.

# **X-ray crystallographic analysis**

**Table S5**. Single crystal X-ray diffraction data.

|  | 4a | 4b | 4c |  |
| --- | --- | --- | --- | --- |
| Chem. Formula | C_37_H_21_N | C_37_H_21_N | C_38_H_22_ |  |
| Form. Wght  [g mol^-1^] | 479.55 | 479.55 | 478.55 |  |
| Crsyt. description | orange  block | yellow  needle | yellow  plate |  |
| Crsyt. system | triclinic | monoclinic | triclinic |  |
| Space group  (Hall group) | P -1  (-P 1) | P 2_1_/n  (-P 2yn) | P -1  (-P 1) |  |
| T [K] | 123 | 123 | 123 |  |
| *a* [Å] | 8.9760(5) | 14.746(2) | 12.5952(13) |  |
| *b* [Å] | 12.0658(6) | 18.515(3) | 12.8054(13) |  |
| *c* [Å] | 22.3014(11) | 17.219(2) | 13.7246(15) |  |
| α [°] | 96.468(2) | 90 | 67.071(4) |  |
| β [°] | 94.220(2) | 90.390(6) | 87.912(4) |  |
| γ [°] | 101.685(2) | 90 | 63.697(4) |  |
| V [Å^3^] | 2338.6(2) | 4701.1(11) | 1802.6(3) |  |
| Z | 4 | 8 | 3 |  |
| *ρ*_calcd_ [g cm^-3^] | 1.362 | 1.355 | 1.323 |  |
| *µ* [mm^-1^] | 0.078 | 0.078 | 0.075 |  |
| *θ*_max_ [°] | 30.999 | 26.999 | 27.000 |  |
| *F*(000) | 1000.0 | 2000.0 | 750.0 |  |
| *h*,*k*,*l*_max_ | 13,17,32 | 18,23,21 | 16,16,17 |  |
| *T_min_,T_max_* | 0.716, 0.746 | 0.621, 0.746 | 0.701, 0.746 |  |
| *N*_ref_ | 14889 | 10265 | 7852 |  |
| *N*_par_ | 685 | 727 | 661 |  |
| λ_MoK\α_ [A] | 0.71073 | 0.71073 | 0.71073 |  |
| R (reflections) | 0.0518  (11807) | 0.0713  (8339) | 0.0567  (6381) |  |
| w*R_2_* (reflections) | 0.1438  (14889) | 0.2053  (10265) | 0.1367  (7852) |  |
| *S* | 1.043 | 1.076 | 1.117 |  |


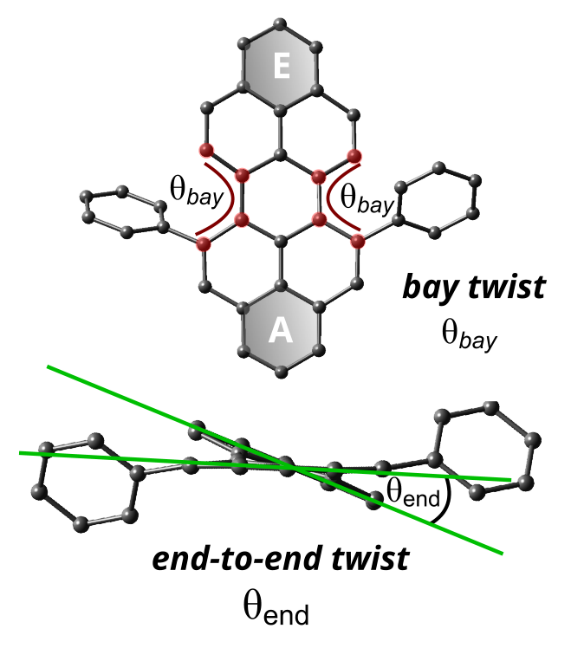


**Figure S1**. Definition of the *bay* (θ_bay_) and end-to-end (θ_end_) twist angles.

All *bay* (θ_bay_) and end-to-end (θ_end_) twist angles reported and referenced in this work were determined using the dihedral angle defined by the four carbon atoms (highlighted in red) within the *bay* regions, and the angle between the mean planes of rings A and E, respectively (Figure S1).

**Table S6**. *Bay* (θ_bay_) and end-to-end (θ_end_) twist angles of DFT ^a^ calculated structures

| Structure | θ_bay_ (°) | θ_end_ (°) |
| --- | --- | --- |
| (*P,P*)-4a | 18.9 | 19.8 |
| (*P,P*)-4b | 19.0 | 19.6 |
| (*P,P*)-4c | 19.3 | 20.1 |

^a^ Calculated at the B3LYP-D3(BJ)/6-311G(d,p) level of theory.

# **Electronic structure and Aromaticity**

## Bond length analysis

**Table S7**. Selected bond lengths in Angstrom (Å) from X-ray crystallographic analysis.

| Structure | a/a' | b/b' | c/c' | d/d' |
| --- | --- | --- | --- | --- |
| 4c | 1.356(3)/1.361(3) | 1.349(3)/1.354(3) | 1.458(3)/1.459(3) | 1.416(3)/1.419(3) |
| 4a (A)^a^ | 1.367(2)/1.368(2) | 1.357(2)/1.357(2) | 1.454(2)/1.457(2) | 1.424(2)/1.425(2) |
| 4a (B)^a^ | 1.367(1)/1.367(1) | 1.357(1)/1.355(1) | 1.457(1)/1.456(1) | 1.426(1)/1.425(1) |
| 4b (A)^a^ | 1.351(4)/1.349(4) | 1.340(4)/1.352(4) | 1.461(4)/1471(4) | 1.411(4)/1.405(4) |
| 4b (B)^a^ | 1.351(4)/1.357(4) | 1.345(4)/1.346(4) | 1.464(4)/1.467(4) | 1.418(4)/1.420(4) |
|  |  |  |  |  |
| Average bond length | 1.359 | 1.351 | 1.460 | 1.419 (≈1.42) |

^a^ Two symmetry-independent structures (A) and (B).

**Table S8**. Average bond length and bond length alternation (BLA) of

the central ring (C) determined from X-ray crystal structures and DFT

optimized geometries.

| Structure | Average bond length (Å) | BLA |  |
| --- | --- | --- | --- |
| PP (exp.)^b^ | 1.4228 | 0.001 |  |
| PP (calc.)^a^ | 1.4250 | 0.000 |
| 2-APP (calc.)^a^ | 1.4246 | 0.000 |
| 1-APP (calc.)^a^ | 1.4246 | 0.000 |
|  |  |  |
| 4c (exp.) | 1.4268 | 0.001 |
| 4c (calc.)^a^ | 1.4273 | 0.000 |
| 4a (A) (exp.)^c^ | 1.4266 | 0.002 |
| 4a (B) (exp.)^c^ | 1.4278 | 0.001 |
| 4a (calc.)^a^ | 1.4276 | 0.000 |
| 4b (A) (exp.)^c^ | 1.4258 | 0.004 |
| 4b (B) (exp.)^c^ | 1.4298 | 0.001 |
| 4b (calc.)^a^ | 1.4275 | 0.000 |
| Average bond length (exp.) | 1.4273 |  |  |

^a^ Calculated at the B3LYP-D3(BJ)/6-311G(d,p) level of theory.

^b^ CCDC Number: 1865911. Ref.[7]

^c^ Two symmetry-independent structures (A) and (B).

## HOMA(c)

**Table S9**. HOMA and HOMAc values determined from bond lengths of

X-ray crystal structures.

| Ring | PP^a^ | 4a (A)^b^ | 4a (B)^b^ | 4b (A)^b^ | 4b (B)^b^ | 4c |  |
| --- | --- | --- | --- | --- | --- | --- | --- |
| A | **0.892 (0.95)** | **0.939 (0.977)** | **0.932 (0.973)** | **0.858 (0.928)** | **0.858 (0.934)** | **0.900 (0.952)** |  |
| B | 0.516 (0.78) | 0.589 (0.79) | 0.555 (0.768) | 0.484 (0.727) | 0.426 (0.693) | 0.494 (0.735) |  |
| B' | 0.581 (0.744) | 0.546 (0.767) | 0.548 (0.771) | 0.379 (0.669) | 0.431 (0.700) | 0.478 (0.728) |  |
| C | **0.680 (0.85)** | **0.609 (0.812)** | **0.587 (0.801)** | **0.587 (0.798)** | **0.520 (0.763)** | **0.590 (0.801)** |  |
| D | 0.581 (0.758) | 0.560 (0.773) | 0.562 (0.791) | 0.440 (0.701) | 0.524 (0.748) | 0.550 (0.765) |  |
| D' | 0.536 (0.779) | 0.586 (0.787) | 0.594 (0.773) | 0.489 (0.733) | 0.473 (0.721) | 0.584 (0.785) |  |
| E | **0.880 (0.941)** | **0.882 (0.946)** | **0.894 (0.953)** | **0.853 (0.93)** | **0.891 (0.951)** | **0.894 (0.949)** |  |

^a^ CCDC Number: 1865911, Ref.[7].

^b^ Two symmetry-independent structures (A) and (B).

HOMAc values are given in parenthesis.

**Table S10**. HOMA and HOMAc values determined from bond lengths of

DFT (B3LYP-D3(BJ)/6-311G(d,p) optimized structures.

| Ring | PP^a^ | PP | 2-APP | 1-APP | 4a | 4b | 4c |  |
| --- | --- | --- | --- | --- | --- | --- | --- | --- |
| A | **0.892 (0.95)** | **0.871 (0.942)** | **0.905** | **0.897** | **0.914 (0.965)** | **0.908 (0.957)** | **0.883 (0.948)** |  |
| B | 0.516 (0.78) | 0.595 (0.792) | 0.632 | 0.629 | 0.574 (0.783) | 0.573 (0.782) | 0.547 (0.768) |  |
| B' | 0.581 (0.744) | 0.595 (0.792) | 0.632 | 0.618 | 0.574 (0.783) | 0.562 (0.776) | 0.547 (0.768) |  |
| C | **0.680 (0.85)** | **0.644 (0.831)** | **0.647** | **0.651** | **0.593 (0.804)** | **0.595 (0.805)** | **0.590 (0.802)** |  |
| D | 0.581 (0.758) | 0.596 (0.793) | 0.593 | 0.594 | 0.591 (0.790) | 0.596 (0.792) | 0.597 (0.793) |  |
| D' | 0.536 (0.779) | 0.597 (0.793) | 0.593 | 0.596 | 0.591 (0.790) | 0.596 (0.793) | 0.596 (0.793) |  |
| E | **0.880 (0.941)** | **0.871 (0.942)** | **0.869** | **0.873** | **0.874 (0.943)** | **0.875 (0.944)** | **0.874 (0.943)** |  |

^a^ X-ray crystal structure, CCDC Number: 1865911, Ref.[7].

HOMAc values are given in parenthesis.

**Table S11**. Differences in HOMA and HOMAc values between selected rings in

X-ray crystal structures and calculated^a^ geometries.

|  | X-ray structures | calculated structures |
| --- | --- | --- |
| ΔHOMA (C−B/B′) | 0.089 | 0.040 |
| ΔHOMAc (C−B/B′) | 0.060 | 0.027 |
| ΔHOMA (C−D/D′) | 0.042 | 0.001 |
| ΔHOMAc (C−D/D′) | 0.038 | 0.012 |
| ΔHOMA (A−B/B′) | 0.404 | 0.339 |
| ΔHOMAc (A−B/B′) | 0.281 | 0.180 |
| ΔHOMA (A−D/D′) | 0.361 | 0.307 |
| ΔHOMAc (A−D/D′) | 0.195 | 0.165 |
| ΔHOMA (E−B/B′) | 0.390 | 0.311 |
| ΔHOMAc (E−B/B′) | 0.221 | 0.167 |
| ΔHOMA (E−D/D′) | 0.347 | 0.280 |
| ΔHOMAc (E−D/D′) | 0.188 | 0.152 |

^a^ Calculated at the B3LYP-D3(BJ)/6-311G(d,p) level of theory.

## NICS

**Table S12**. NICS(1.7)_zz_ values in ppm.

|  | A | B | B’ | C | D | D’ | E |
| --- | --- | --- | --- | --- | --- | --- | --- |
| **PP** | -26 | -18 | -18 | -28 | -18 | -18 | -26 |
| **2-APP** | -25 | -17 | -17 | -28 | -18 | -18 | -26 |
| **1-APP** | -24 | -18 | -18 | -27 | -17 | -17 | -25 |
| **4c*^a^*** | -25 | -17 | -17 | -27 | -17 | -17 | -25 |
| **4c**(exp.)***^a,b^*** | -25 | -17 | -17 | -28 | -17 | -17 | -26 |
| **4a*^a^*** | -24 | -16 | -16 | -27 | -17 | -17 | -25 |
| **4a**(exp.)***^a,b,c^*** (A) | -24 | -15 | -18 | -27 | -16 | -16 | -26 |
| **4a**(exp.)***^a,b,c^***  (B) | -24 | -16 | -16 | -25 | -18 | -17 | -26 |
| **4b*^a^*** | -23 | -16 | -17 | -27 | -17 | -17 | -25 |
| **4b**(exp.)***^a,b,c^*** (A) | -23 | -18 | -15 | -28 | -17 | -17 | -26 |
| **4b**(exp.)***^a,b,c^*** (B) | -24 | -17 | -16 | -26 | -17 | -17 | -26 |

*^a^* NICS(1.7)_zz_-avg. values. *^b^* Non-optimized experimental geometry.*^c^* Two symmetry independent structures (A) and (B).

**Table S13**. NICS(1.7)_πzz_ values in ppm.

|  | A | B | B’ | C | D | D’ | E |
| --- | --- | --- | --- | --- | --- | --- | --- |
| **PP** | -22 | -13 | -13 | -24 | -13 | -13 | -22 |
| **2-APP** | -21 | -13 | -13 | -24 | -13 | -13 | -22 |
| **1-APP** | -20 | -13 | -14 | -24 | -13 | -13 | -22 |
| **4c*^a^*** | -20 | -12 | -12 | -22 | -12 | -12 | -20 |
| **4a*^a^*** | -18 | -12 | -12 | -22 | -12 | -12 | -20 |
| **4b*^a^*** | -18 | -12 | -13 | -23 | -12 | -12 | -20 |

*^a^* NICS(1.7)_πzz_-avg. values

## NICS2BC


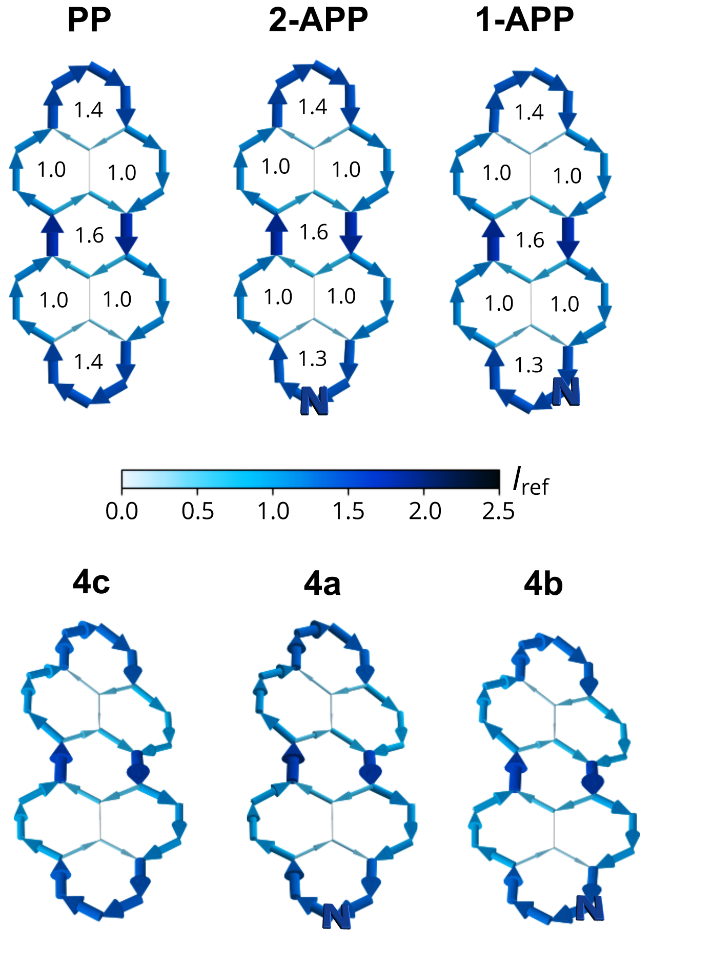


**Figure S2.** NICS2BC bond current graphs of planar (A)PPs with corresponding ring weight values (top) and twisted (A)PPs **4a-c** (bottom). Phenyl substituents are not shown. Bond current strength relative to *I*_ref_ (bond current of benzene, 11.5 nA T^-1^).

## ACID-π


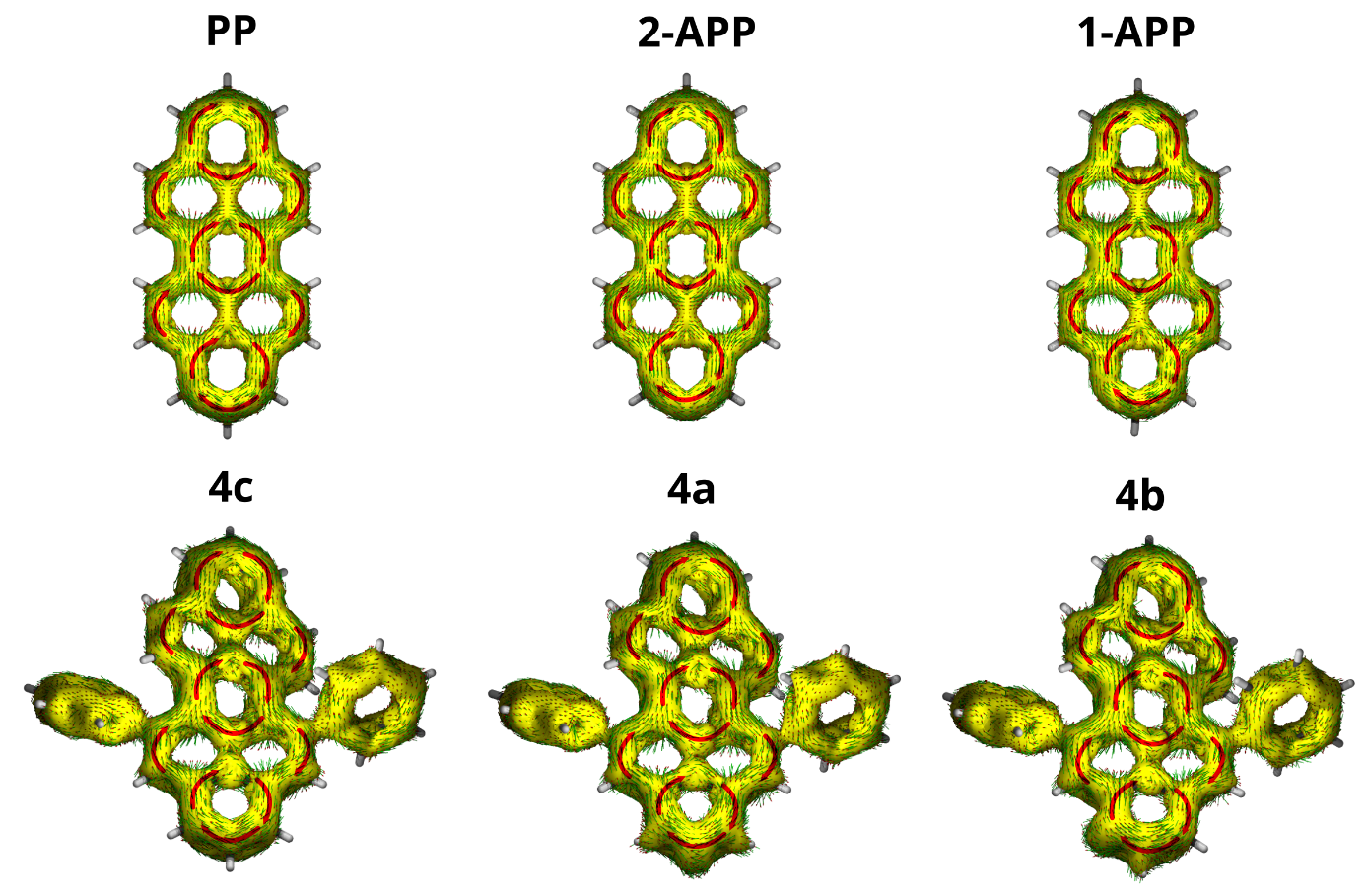


**Figure S3.** ACID-π plots of planar (top) and twisted (bottom) (A)PPs (isovalue = 0.03 a.u.).

## LOL-π maps


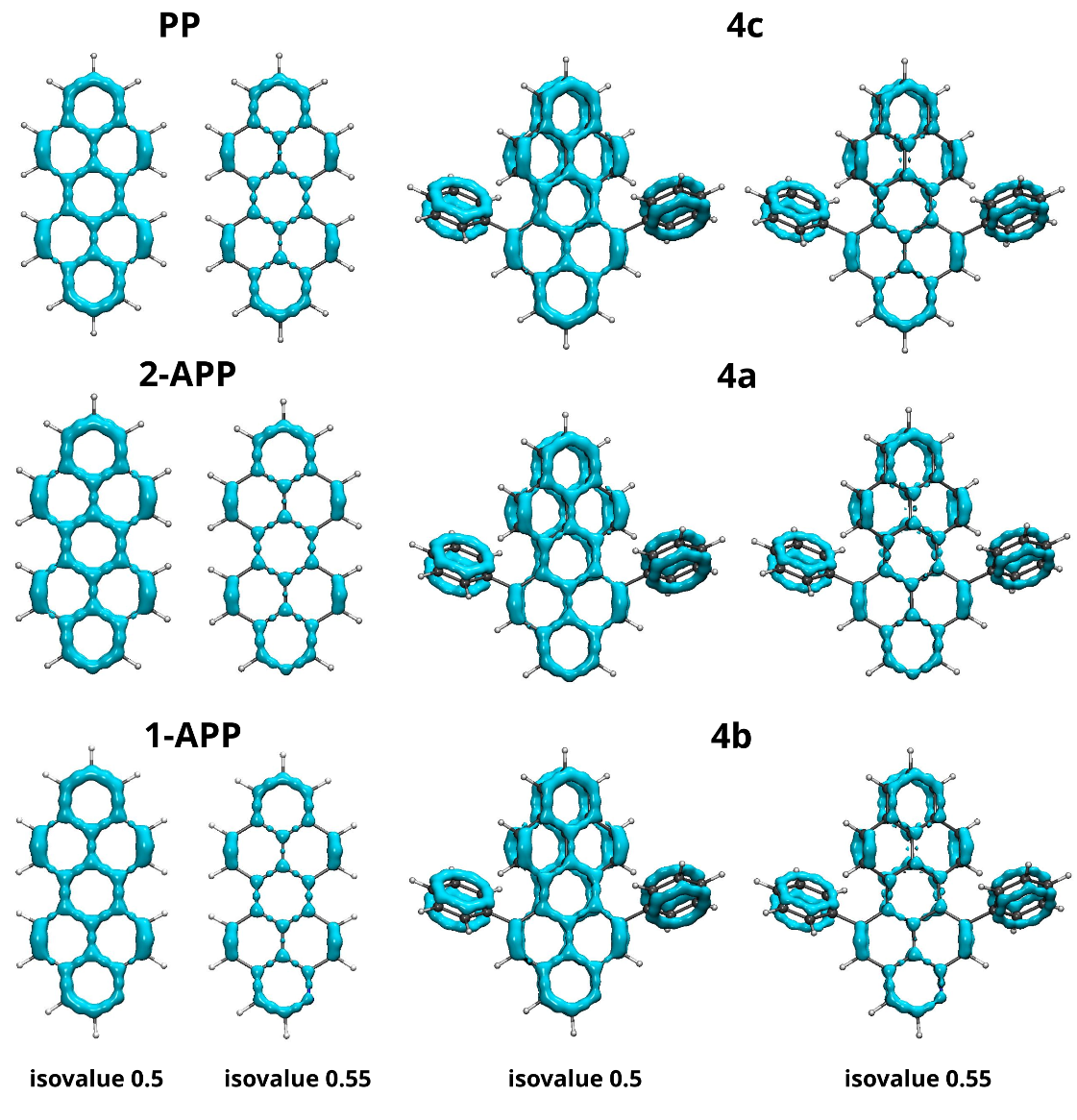


**Figure S4.** LOL-π maps of planar and twisted (A)PPs at different isosurface values.


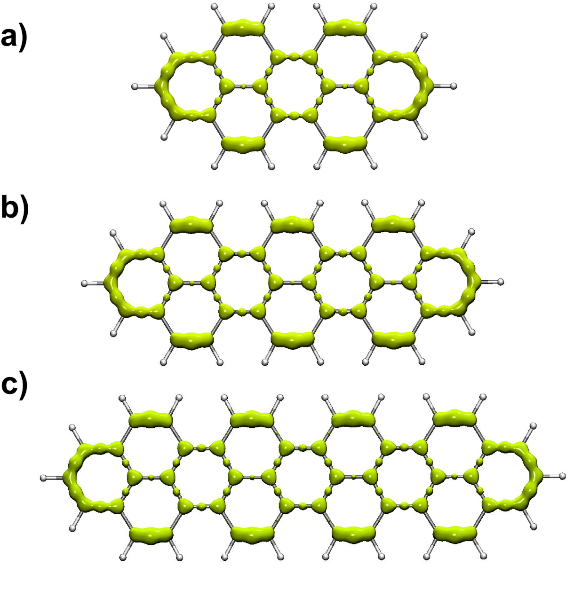


**Figure S5.** LOL-π maps of a) peropyrene, b) terropyrene and c) quaterropyrene

(isosurface values = 0.55 a.u.).

## Multicenter Bond order (MCBO)

**Table S14**. Normalized multi-center bond order (MCBO-π) values.

| Ring | 4c | 4a | 4b |
| --- | --- | --- | --- |
| A | **0.598** | **0.597** | **0.596** |
| B | 0.516 | 0.515 | 0.517 |
| B' | 0.520 | 0.519 | 0.519 |
| C | **0.553** | **0.554** | **0.553** |
| D | 0.520 | 0.520 | 0.520 |
| D' | 0.519 | 0.519 | 0.519 |
| E | **0.600** | **0.600** | **0.600** |

## AV1245

**Table S15**. AV1245 values.

| Ring | 4c | 4a | 4b |
| --- | --- | --- | --- |
| A | **8.79** | **8.57** | **8.10** |
| B | 3.12 | 3.08 | 3.11 |
| B' | 3.13 | 3.09 | 3.07 |
| C | **5.32** | **5.32** | **5.26** |
| D | 3.20 | 3.20 | 3.22 |
| D' | 3.21 | 3.21 | 3.23 |
| E | **8.98** | **8.98** | **8.97** |


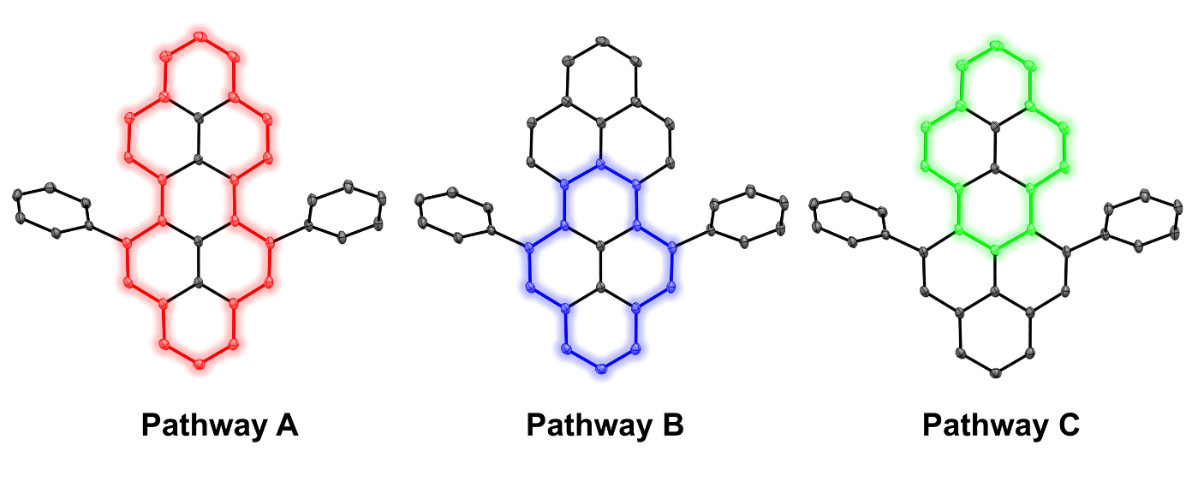


**Figure S6.** AV1245 analysis of electron delocalization pathways.

**Table S16**. AV1245 values for different

electron delocalization pathways.

| Pathway | 4c | 4a | 4b |
| --- | --- | --- | --- |
| A | 2.93 | 2.90 | 2.85 |
| B | 3.11 | 3.08 | 2.97 |
| C | 3.13 | 3.14 | 3.13 |

# **Photophysical Data**

**Table S17**. Photophysical data in toluene at 20 °C (c ≈ 10^-5^ M).

| Compd. | λ_abs_^(max)^ (nm) | ε   (10^4^ M^-1^ cm^-1^) | λ_em_^(max)^ (nm) | Strokes shift (cm^-1^) | Φ*_F_ ^a^* | *τ ^b^* (ns) | *τ_0_ ^c^* (ns) | *k*_r_ ^d^  (10^7^ s^-1^) | *k*_nr_ ^e^  (10^7^ s^-1^) |
| --- | --- | --- | --- | --- | --- | --- | --- | --- | --- |
| 4c | 462 | 5.32 | 478 | 724 | 0.71 | 2.58 | 3.63 | 27.5 | 11.2 |
| 4a | 461 | 6.14 | 477 | 728 | 0.73 | 2.80 | 3.83 | 26.1 | 9.6 |
| 4b | 468 | 4.86 | 485 | 749 | 0.69 | 2.88 | 4.17 | 23.9 | 10.7 |

^a^ Determined relative to coumarin 153 in ethanol (Φ*_F_* = 0.4, λ_ex_. = 420 nm). ^b^ Measured in air. ^c^ Natural lifetime (*τ*/Φ*_F_* ). ^d^ Radiative decay rate constant (Φ*_F_*/*τ*). ^e^ Non-radiative decay rate constant (1/*τ*-(Φ*_F_/τ*)).

**Figure S7**. Fluorescence decay curves in toluene.

# **Electrochemical Data**

**Table S18**. Cyclic voltammetry (CV) and differential pulse voltammetry (DPV) measurements in CH_2_Cl_2_ with 0.1 M *n*Bu_4_NPF_6_ at room temperature. c ≈ 10^-3^ M, scan rate = 100 mV s^-1^.

| Compound | *E*^(ox1)^ [V] | | *E*^(ox2)^ [V] | *E*^(ox3)^ [V] | *E*(*^ox4)^* [V] | *E*^(red1)^ [V] |
| --- | --- | --- | --- | --- | --- | --- |
| 4c | CV | 0.56^a^ (0.56) | 1.10^b^ | 1.59^b^ | - | -1.95^a^  (-1.95) |
|  | DPV | 0.54 | 1.10 | 1.59 | - | -1.97 |
| 4a | CV | 0.63^b^ | 0.86^b^ | 1.04^b^ | 1.32^b^ | -1.86^a^  (-1.86) |
|  | DPV | 0.62 | 0.84 | 1.02 | 1.31 | -1.88 |
| 4b | CV | 0.75^b^ | 1.20^c^ | 1.52^b^ | - | -1.73^a^  (-1.73) |
|  | DPV | 0.74 | 1.18 | - | - | -1.75 |

All CV redox values are estimates of *E*^(0)^ determined from the inflection points of the cyclic voltammograms.^[9]^ Half-wave potentials (*E*^(1/2)^) are given in parentheses. ^a^ reversible, ^b^ irreversible, ^c^ partially reversible.

# **Computational Studies**

All calculations were performed with Gaussian 09 (Revision E.01).^[10]^ Geometry optimizations were performed in the gas phase without symmetry constraints with the B3LYP^[11]^ global-hybrid GGA functional and Pople’s^[12]^ split-valence triple-ζ basis set (6-311G), including Grimme’s^[13]^ D3 dispersion correction with Becke–Johnson^[14]^ (BJ) damping. Harmonic vibration frequency calculations were performed to verify all stationary points as local minima (with no imaginary frequency) or transition states as first-order saddle-points (with one imaginary frequency) on the potential energy surface.

Time-dependent density functional theory (TDDFT) calculations were performed at the same level of theory using the the Tamm-Dancoff^[15]^ approximation (TDA). Solvent effects were included using the polarizable continuum model (PCM) in its integral equation formalism variant (IEFPCM).

Nucleus independent chemical shift^[16]^ NICS(1.7)_zz_ values were calculated with the gauge-including atomic orbital (GIAO) method at the B3LYP/6-311+G(d,p) level of theory. NICS(1.7)_πzz_ values were calculated with the continuous set of gauge transformations (CSGT) method at the same level of theory with contribution from π-orbitals only. NICS(1.7)_zz_-avg. and NICS(1.7)_πzz_-avg. values correspond to the average taken 1.7 Å above and below the geometric centers normal to the ring planes. The unit normal vector perpendicular to the ring plane to obtain the shielding values normal to the planes was calculated with the multifunctional wavefunction analyzer Multiwfn^[17]^ software (3.8). The NICS(1.7) and NICS(1.7)_πzz_ metric is used as recommended in Refs.^[18]^ NICS values were rounded to the nearest whole number and expressed in parts per million (ppm). NICS2BC^[19]^ bond currents graphs were generated from NICS(1.25)_zz_ values calculated using the GIAO method at the B3LYP/6-311+G(d,p) level and generated with the BC-Wizard. Anisotropy of the Induced Current Density^[20]^ (ACID) calculations were performed with the CSGT method at the B3LYP/6-311G(d,p) level. ACID plots with contribution from π-orbitals only were generated with the ACID program (3.0.4) at 0.03 a.u. isosurface. The Localized orbital locator (LOL)-π isosurface maps^[21]^, normalized multicenter bond order (MCBO-π), AV1245 and HOMA(c) were calculated with Multiwfn. LOL isosurface maps were visualized with the VMD^[22]^ program.


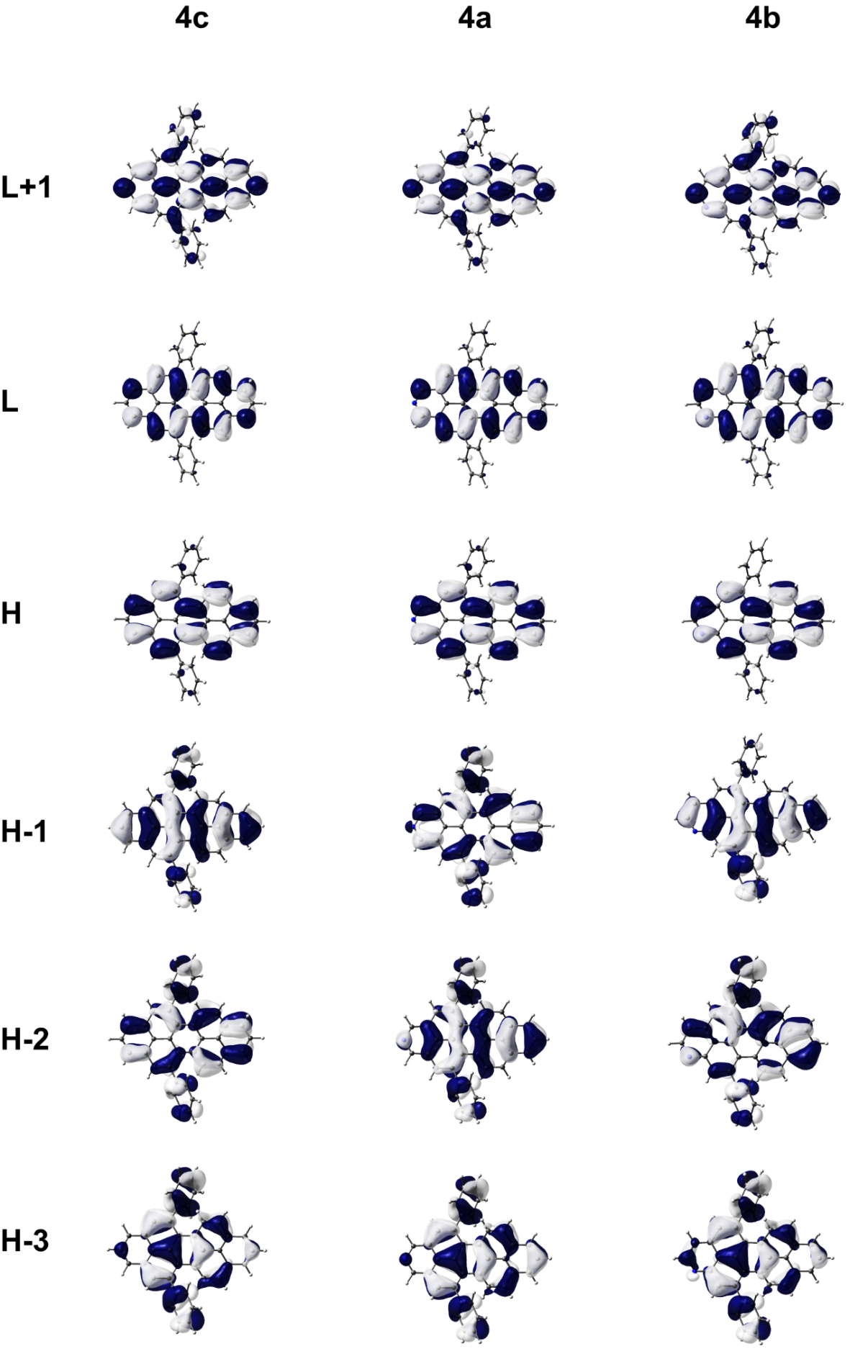


**Figure S8.** Molecular orbitals of peropyrenes **4** calculated at the (B3LYP-D3(BJ)/6-311G(d,p) level of theory (isovalue = 0.02 a.u.).


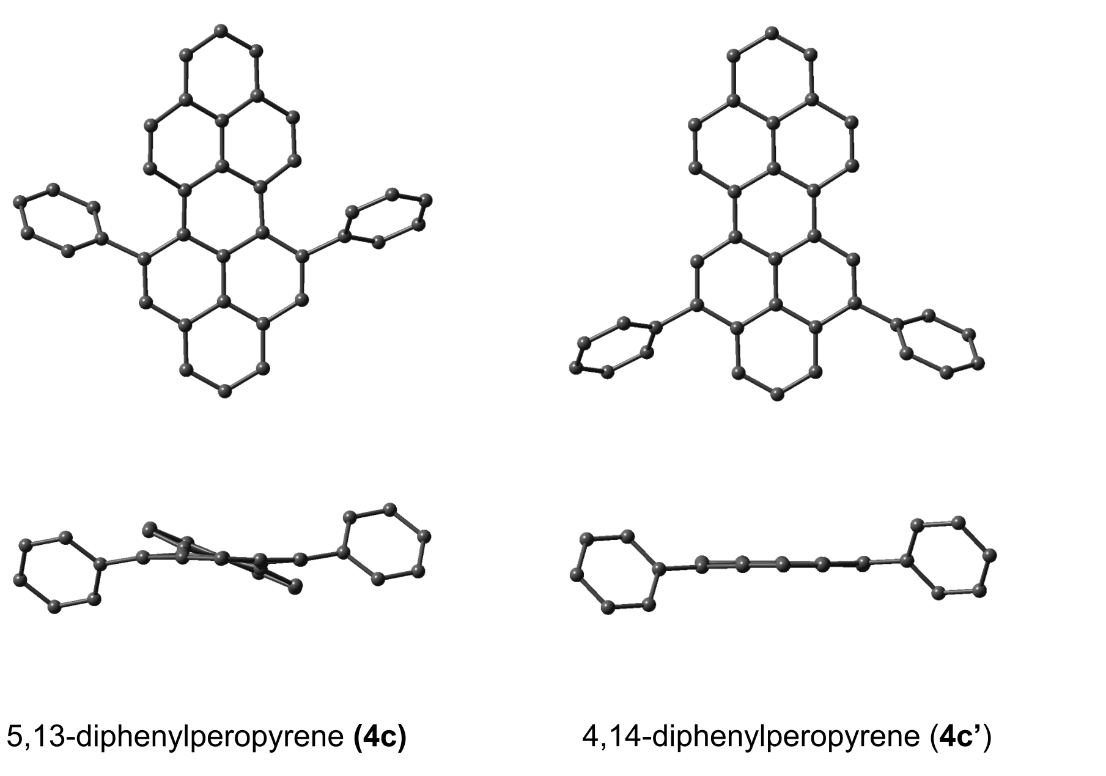


**Figure S9.** Comparsion of the optimized ground-state structures of positional isomers **4c** and **4c’** in top and side views calculated at the B3LYP-D3(BJ)/6-311G(d,p) level of theory.

**Table S19.** TDA-DFT (B3LYP/6-311G(d,p) calculated singlet excitation energies,

oscillator strengths (*f*) and main configurations of (*P*,*P*)-**4a** in toluene.

| S_n_ | *E*(eV) | λ(nm) | *f* | Configuration (Major Contributions > 10%) |
| --- | --- | --- | --- | --- |
| 1 | 2.7002 | 459.17 | 1.09280 | H → L 93.4% |
| 2 | 2.9875 | 415.01 | 0.03240 | H → L+1 70.1%  H-2 → L 24.7% |
| 3 | 3.3341 | 371.87 | 0.00300 | H-1 → L 67.1%  H → L+2 31.8% |
| 4 | 3.4545 | 358.91 | 0.22160 | H-2 → L 63.6%  H-3 → L 19.7%  H → L+1 13.2% |
| 5 | 3.5717 | 347.13 | 0.02030 | H → L+3 75.1%  H-3 → L 19.6% |
| 6 | 3.7303 | 332.37 | 0.00480 | H → L+2 56.5%  H-1 → L 23.6% |

**Table S20.** TDA-DFT (B3LYP/6-311G(d,p) calculated singlet excitation energies,

oscillator strengths (*f*) and main configurations of (*P*,*P*)-**4b** in toluene.

| S_n_ | E(eV) | λ(nm) | *f* | Configuration (Major Contributions > 10%) |
| --- | --- | --- | --- | --- |
| 1 | 2.6942 | 460.19 | 1.06630 | H → L 92.8% |
| 2 | 3.0304 | 409.13 | 0.02030 | H-1 → L 57.5%  H → L+1 34.9% |
| 3 | 3.3173 | 373.75 | 0.02960 | H-2 → L 48.2% H-1 → L 15.7%  H → L+2 14.5%  H → L+1 14.2% |
| 4 | 3.4223 | 362.28 | 0.11650 | H-3 → L 52.4% H-2 → L 18.6%  H → L+1 10.3% |
| 5 | 3.6149 | 342.98 | 0.13050 | H → L+3 37.4%  H → L+1 24.6% H-3 → L 16.9% |
| 6 | 3.7522 | 330.43 | 0.02270 | H → L+2 61.1% H-2 → L 13.0% |

**Table S21.** TDA-DFT (B3LYP/6-311G(d,p) calculated singlet excitation energies,

oscillator strengths (*f*) and main configurations of (*P*,*P*)-**4c** in toluene.

| S_n_ | *E*(eV) | λ(nm) | *f* | Configuration (Major Contributions > 10%) |
| --- | --- | --- | --- | --- |
| 1 | 2.6857 | 461.65 | 1.11590 | H → L 93.6% |
| 2 | 3.0086 | 412.10 | 0.00200 | H → L+1 55.4%  H-1 → L 42.7% |
| 3 | 3.3392 | 371.30 | 0.00840 | H-2 → L 60.1%  H → L+2 38.9% |
| 4 | 3.4752 | 356.77 | 0.26470 | H-1 → L 41.3%  H-3 → L 26.0%  H → L+1 25.3% |
| 5 | 3.5481 | 349.44 | 0.00560 | H → L+3 68.8%  H-3 → L 27.4% |
| 6 | 3.7325 | 332.17 | 0.00450 | H → L+2 53.6%  H-2 → L 32.5% |

**Table S22.** TDA-DFT (B3LYP/6-311G(d,p) calculated singlet excitation energies,

oscillator strengths (*f*) and main configurations of (*P*,*P*)-**4a-H^+^** in toluene.

| S_n_ | *E*(eV) | λ(nm) | *f* | Configuration (Major Contributions > 10%) |
| --- | --- | --- | --- | --- |
| 1 | 2.4490 | 507.11 | 0.06770 | H → L+1 91.6% |
| 2 | 2.5024 | 495.46 | 0.71930 | H → L 86.9% |
| 3 | 3.0532 | 406.08 | 0.18000 | H-1 → L 91.9% |
| 4 | 3.1510 | 393.48 | 0.00970 | H-2 → L 90.1% |
| 5 | 3.3198 | 373.47 | 0.21250 | H-1 → L+1  89.4% |
| 6 | 3.3346 | 371.81 | 0.06810 | H-2 → L+1  93.3% |

**Table S23.** TDA-DFT (B3LYP/6-311G(d,p) calculated singlet excitation energies,

oscillator strengths (*f*) and main configurations of (*P*,*P*)-**4b-H^+^** in toluene.

| S_n_ | E(eV) | λ(nm) | *f* | Configuration (Major Contributions > 10%) |
| --- | --- | --- | --- | --- |
| 1 | 2.3800 | 520.94 | 0.89880 | H → L 90.9% |
| 2 | 2.7140 | 456.83 | 0.19160 | H-1 → L 93.1% |
| 3 | 2.9637 | 418.34 | 0.12290 | H-2 → L 91.4% |
| 4 | 3.0692 | 403.96 | 0.03310 | H-3 → L 79.2%  H → L+1 13.5% |
| 5 | 3.1800 | 389.89 | 0.01750 | H-4 → L 88.3% |
| 6 | 3.2684 | 379.34 | 0.00260 | H-5 → L 96.0% |

**Cartesian Coordinates**

| **PP (S_0_)** | | | |
| --- | --- | --- | --- |
| Charge = 0, Multiplicity = 1 | | | |
| Number of imaginary frequencies = 0 | | | |
| Electronic Energy = -999.619608631 Hartree | | | |
| Sum of electronic and zero-point Energies = -999.306732 Hartree | | | |
|  | | | |
| Atoms | Cartesian Coordinates | | |
|  | **X** | **Y** | **Z** |
| C | 1.204834 | 4.973463 | 0.000000 |
| C | 1.226745 | 3.571511 | 0.000000 |
| C | -0.000019 | 2.848324 | 0.000000 |
| C | -1.226856 | 3.571531 | 0.000000 |
| C | 2.449930 | 2.832426 | 0.000000 |
| C | -0.000019 | 1.416818 | 0.000000 |
| C | 1.240018 | 0.710700 | 0.000000 |
| C | 2.452869 | 1.474752 | 0.000000 |
| C | -1.240031 | 0.710665 | 0.000000 |
| C | -2.452934 | 1.474711 | 0.000000 |
| C | -2.450056 | 2.832356 | 0.000000 |
| H | -3.385961 | 3.380279 | 0.000000 |
| H | 2.145108 | 5.513221 | 0.000000 |
| C | 1.240009 | -0.710686 | 0.000000 |
| C | 0.000028 | -1.416747 | 0.000000 |
| C | -1.239981 | -0.710691 | 0.000000 |
| C | 2.452948 | -1.474746 | 0.000000 |
| C | 0.000037 | -2.848383 | 0.000000 |
| C | 1.226901 | -3.571546 | 0.000000 |
| C | 2.450211 | -2.832369 | 0.000000 |
| C | 1.204864 | -4.973380 | 0.000000 |
| H | 2.145205 | -5.513327 | 0.000000 |
| C | 0.000058 | -5.665741 | 0.000000 |
| C | -1.204763 | -4.973389 | 0.000000 |
| C | -1.226818 | -3.571563 | 0.000000 |
| C | -2.450148 | -2.832403 | 0.000000 |
| C | -2.452912 | -1.474785 | 0.000000 |
| H | -3.385994 | -3.380342 | 0.000000 |
| H | 3.386068 | -3.380289 | 0.000000 |
| H | 0.000064 | -6.749510 | 0.000000 |
| H | -2.145096 | -5.513349 | 0.000000 |
| H | 3.403459 | -0.961076 | 0.000000 |
| H | -3.403432 | -0.961136 | 0.000000 |
| H | -3.403532 | 0.961238 | 0.000000 |
| H | 3.403484 | 0.961309 | 0.000000 |
| H | 3.385837 | 3.380357 | 0.000000 |
| C | 0.000037 | 5.665811 | 0.000000 |
| H | -0.000139 | 6.749631 | 0.000000 |
| C | -1.204932 | 4.973319 | 0.000000 |
| H | -2.145189 | 5.513260 | 0.000000 |

| **2-APP (S_0_)** | | | |
| --- | --- | --- | --- |
| Charge = 0, Multiplicity = 1 | | | |
| Number of imaginary frequencies = 0 | | | |
| Electronic Energy = -1015.65617238 Hartree | | | |
| Sum of electronic and zero-point Energies = -1015.354973 Hartree | | | |
|  | | | |
| Atoms | Cartesian Coordinates | | |
|  | **X** | **Y** | **Z** |
| C | 1.204723 | -4.965579 | 0.000000 |
| C | 1.226966 | -3.563814 | 0.000000 |
| C | -0.000001 | -2.840328 | 0.000000 |
| C | -1.226976 | -3.563820 | 0.000000 |
| C | 2.450946 | -2.826219 | 0.000000 |
| C | -0.000001 | -1.408878 | 0.000000 |
| C | 1.242552 | -0.704567 | 0.000000 |
| C | 2.454981 | -1.468582 | 0.000000 |
| C | -1.242552 | -0.704562 | 0.000000 |
| C | -2.454986 | -1.468580 | 0.000000 |
| C | -2.450955 | -2.826212 | 0.000000 |
| H | -3.386260 | -3.374918 | 0.000000 |
| H | 2.144948 | -5.505592 | 0.000000 |
| C | 1.243206 | 0.716471 | 0.000000 |
| C | 0.000002 | 1.413541 | 0.000000 |
| C | -1.243206 | 0.716469 | 0.000000 |
| C | 2.453341 | 1.489451 | 0.000000 |
| C | 0.000003 | 2.840444 | 0.000000 |
| C | 1.214982 | 3.571644 | 0.000000 |
| C | 2.446688 | 2.848506 | 0.000000 |
| C | 1.141679 | 4.971934 | 0.000000 |
| H | 2.058712 | 5.555767 | 0.000000 |
| C | -1.141671 | 4.971934 | 0.000000 |
| C | -1.214975 | 3.571648 | 0.000000 |
| C | -2.446683 | 2.848509 | 0.000000 |
| C | -2.453340 | 1.489455 | 0.000000 |
| H | -3.378985 | 3.402044 | 0.000000 |
| H | 3.378990 | 3.402039 | 0.000000 |
| H | -2.058702 | 5.555770 | 0.000000 |
| H | 3.405650 | 0.978972 | 0.000000 |
| H | -3.405649 | 0.978978 | 0.000000 |
| H | -3.405420 | -0.954748 | 0.000000 |
| H | 3.405418 | -0.954755 | 0.000000 |
| H | 3.386251 | -3.374924 | 0.000000 |
| C | 0.000003 | -5.657867 | 0.000000 |
| H | -0.000011 | -6.741561 | 0.000000 |
| C | -1.204731 | -4.965570 | 0.000000 |
| H | -2.144943 | -5.505605 | 0.000000 |
| N | 0.000005 | 5.660851 | 0.000000 |

| **1-APP (S_0_)** | | | |
| --- | --- | --- | --- |
| Charge = 0, Multiplicity = 1 | | | |
| Number of imaginary frequencies = 0 | | | |
| Electronic Energy = -1015.65879971 Hartree | | | |
| Sum of electronic and zero-point Energies = -1015.357457 Hartree | | | |
|  | | | |
| Atoms | Cartesian Coordinates | | |
|  | **X** | **Y** | **Z** |
| C | 1.163548 | -4.970672 | -0.000000 |
| C | 1.235013 | -3.572285 | -0.000000 |
| C | 0.015185 | -2.846533 | -0.000000 |
| C | -1.201338 | -3.584521 | -0.000000 |
| C | 2.462012 | -2.839412 | -0.000000 |
| C | 0.008298 | -1.418993 | -0.000000 |
| C | 1.247036 | -0.713911 | -0.000000 |
| C | 2.459432 | -1.480506 | -0.000000 |
| C | -1.235113 | -0.723247 | 0.000000 |
| C | -2.442095 | -1.498304 | 0.000000 |
| C | -2.432264 | -2.857002 | -0.000000 |
| H | -3.348780 | -3.433251 | -0.000000 |
| H | 2.068906 | -5.566010 | -0.000000 |
| C | 1.243979 | 0.707089 | -0.000000 |
| C | 0.000000 | 1.408009 | 0.000000 |
| C | -1.238840 | 0.698142 | 0.000000 |
| C | 2.454227 | 1.475395 | -0.000000 |
| C | -0.004856 | 2.839301 | 0.000000 |
| C | 1.219103 | 3.566864 | 0.000000 |
| C | 2.445432 | 2.832916 | 0.000000 |
| C | 1.192234 | 4.968461 | 0.000000 |
| H | 2.130616 | 5.511705 | 0.000000 |
| C | -0.014884 | 5.656854 | 0.000000 |
| C | -1.216994 | 4.959935 | 0.000000 |
| C | -1.233971 | 3.558090 | 0.000000 |
| C | -2.454938 | 2.815435 | 0.000000 |
| C | -2.454288 | 1.457825 | 0.000000 |
| H | -3.392300 | 3.360672 | 0.000000 |
| H | 3.378860 | 3.384879 | 0.000000 |
| H | -0.018739 | 6.740491 | 0.000000 |
| H | -2.159216 | 5.496473 | 0.000000 |
| H | 3.406617 | 0.965231 | -0.000000 |
| H | -3.402424 | 0.940003 | 0.000000 |
| H | -3.396579 | -0.991622 | 0.000000 |
| H | 3.409460 | -0.965465 | -0.000000 |
| H | 3.397690 | -3.386903 | -0.000000 |
| N | -1.244996 | -4.929341 | -0.000000 |
| C | -0.083495 | -5.585720 | -0.000000 |
| H | -0.153668 | -6.670068 | -0.000000 |

| **(*P,P*)-4c (S_0_)** | | | |
| --- | --- | --- | --- |
| Charge = 0, Multiplicity = 1 | | | |
| Number of imaginary frequencies = 0 | | | |
| Electronic Energy = -1461.87587434 Hartree | | | |
| Sum of electronic and zero-point Energies = -1461.401853 Hartree | | | |
| Sum of electronic and thermal Free Energies = -1461.457106 Hartree | | | |
|  | | | |
| Atoms | Cartesian Coordinates | | |
|  | **X** | **Y** | **Z** |
| C | -1.221496 | -3.491996 | 0.085356 |
| C | -1.201805 | -4.894805 | 0.076846 |
| C | 0.000150 | -5.587396 | 0.000104 |
| C | 1.202059 | -4.894737 | -0.076674 |
| C | 1.221664 | -3.491925 | -0.085270 |
| C | 0.000062 | -2.769568 | 0.000017 |
| C | 0.000025 | -1.339789 | -0.000022 |
| C | -1.236723 | -0.633924 | 0.159413 |
| C | -2.473453 | -1.390633 | 0.116308 |
| C | -2.440165 | -2.756470 | 0.105525 |
| C | 1.236740 | -0.633854 | -0.159478 |
| C | 1.187898 | 0.775410 | -0.357162 |
| C | -0.000060 | 1.479431 | -0.000002 |
| C | -1.187965 | 0.775335 | 0.357145 |
| C | 2.440288 | -2.756334 | -0.105491 |
| C | 2.473512 | -1.390493 | -0.116360 |
| C | -0.000097 | 2.910623 | 0.000022 |
| C | -1.124331 | 3.631461 | 0.493218 |
| C | -2.234646 | 2.887034 | 0.998251 |
| C | -2.269032 | 1.531612 | 0.921507 |
| C | 1.124099 | 3.631547 | -0.493137 |
| C | 1.099189 | 5.033410 | -0.494365 |
| C | -0.000172 | 5.725386 | 0.000095 |
| C | -1.099497 | 5.033327 | 0.494518 |
| C | 2.268927 | 1.531779 | -0.921472 |
| C | 2.234464 | 2.887202 | -0.998180 |
| C | -3.805943 | -0.752647 | -0.066295 |
| C | 3.805995 | -0.752467 | 0.066198 |
| C | -4.036396 | 0.106333 | -1.148153 |
| C | -5.291850 | 0.664537 | -1.352667 |
| C | -6.340424 | 0.376342 | -0.479631 |
| C | -6.124319 | -0.483017 | 0.593766 |
| C | -4.866852 | -1.047116 | 0.795974 |
| C | 4.866885 | -1.046911 | -0.796104 |
| C | 6.124356 | -0.482814 | -0.593918 |
| C | 6.340490 | 0.376527 | 0.479487 |
| C | 5.291929 | 0.664715 | 1.352544 |
| C | 4.036477 | 0.106502 | 1.148061 |
| H | -2.141637 | -5.433065 | 0.127525 |
| H | 0.000182 | -6.671173 | 0.000144 |
| H | 2.141926 | -5.432940 | -0.127323 |
| H | -3.372635 | -3.305349 | 0.037904 |
| H | 3.372787 | -3.305164 | -0.037854 |
| H | -3.061513 | 3.426076 | 1.447197 |
| H | -3.122854 | 1.008803 | 1.320490 |
| H | 1.954188 | 5.573358 | -0.885892 |
| H | -0.000198 | 6.809227 | 0.000132 |
| H | -1.954528 | 5.573212 | 0.886061 |
| H | 3.122785 | 1.009035 | -1.320452 |
| H | 3.061302 | 3.426300 | -1.447111 |
| H | -3.221235 | 0.337987 | -1.822874 |
| H | -5.453640 | 1.326487 | -2.195558 |
| H | -7.317708 | 0.817463 | -0.637073 |
| H | -6.932997 | -0.712762 | 1.278171 |
| H | -4.694896 | -1.703658 | 1.641193 |
| H | 4.694918 | -1.703432 | -1.641339 |
| H | 6.933017 | -0.712561 | -1.278341 |
| H | 7.317777 | 0.817647 | 0.636909 |
| H | 5.453729 | 1.326668 | 2.195431 |
| H | 3.221332 | 0.338143 | 1.822809 |

| **(*P,P*)-4c (TS)** | | | |
| --- | --- | --- | --- |
| Charge = 0, Multiplicity = 1 | | | |
| Number of imaginary frequencies = 1, *v*_i_ = -23.26 | | | |
| Electronic Energy = -1461.87102984 Hartree | | | |
| Sum of electronic and thermal Free Energies = -1461.451119 Hartree | | | |
|  | | | |
| Atoms | Cartesian Coordinates | | |
|  | **X** | **Y** | **Z** |
| C | -1.406691 | -3.343525 | -0.437168 |
| C | -0.149075 | -2.693029 | -0.354968 |
| C | 1.030022 | -3.484819 | -0.438800 |
| C | 0.930010 | -4.868387 | -0.643099 |
| C | -2.573351 | -2.556271 | -0.245286 |
| C | -0.072912 | -1.269315 | -0.209700 |
| C | -1.273686 | -0.483306 | -0.096217 |
| C | -2.535499 | -1.207830 | -0.035733 |
| C | 1.219271 | -0.651787 | -0.212432 |
| C | 2.393526 | -1.497450 | -0.062910 |
| C | 2.283450 | -2.847958 | -0.219164 |
| H | 3.161593 | -3.470643 | -0.091993 |
| H | -3.533111 | -3.059719 | -0.217125 |
| H | 1.839402 | -5.456085 | -0.699204 |
| C | -1.158260 | 0.944322 | -0.084494 |
| C | 0.124892 | 1.549989 | -0.285637 |
| C | 1.303940 | 0.754008 | -0.394176 |
| C | -2.278982 | 1.832801 | 0.052411 |
| C | 0.238901 | 2.977438 | -0.390834 |
| C | -0.901433 | 3.812160 | -0.228215 |
| C | -2.158498 | 3.183707 | 0.000301 |
| C | -0.769163 | 5.206178 | -0.308929 |
| H | -1.649676 | 5.824116 | -0.172499 |
| C | 0.464540 | 5.788376 | -0.566559 |
| C | 1.584847 | 4.987814 | -0.754380 |
| C | 1.493893 | 3.591744 | -0.670283 |
| C | 2.622940 | 2.749371 | -0.897987 |
| C | 2.528775 | 1.403598 | -0.768484 |
| H | 3.562297 | 3.203312 | -1.193493 |
| H | -3.039219 | 3.805755 | 0.117380 |
| H | 0.552758 | 6.866750 | -0.629736 |
| H | 2.547471 | 5.436659 | -0.972776 |
| H | -3.261362 | 1.431764 | 0.201363 |
| H | 3.395621 | 0.802684 | -0.983835 |
| C | 3.707915 | -0.980874 | 0.406188 |
| C | 3.783756 | -0.201402 | 1.567685 |
| C | 4.893970 | -1.315785 | -0.255960 |
| C | 5.011568 | 0.238633 | 2.045567 |
| H | 2.871123 | 0.061408 | 2.088507 |
| C | 6.124205 | -0.870391 | 0.220861 |
| H | 4.844716 | -1.907181 | -1.163006 |
| C | 6.187390 | -0.090086 | 1.371959 |
| H | 5.052267 | 0.840230 | 2.946289 |
| H | 7.032563 | -1.129239 | -0.311129 |
| H | 7.143961 | 0.259418 | 1.742416 |
| C | -3.866372 | -0.616725 | 0.302154 |
| C | -4.833295 | -0.415246 | -0.685787 |
| C | -4.191167 | -0.344391 | 1.635310 |
| C | -6.091509 | 0.080967 | -0.352132 |
| H | -4.585913 | -0.630709 | -1.718584 |
| C | -5.447790 | 0.149516 | 1.968579 |
| H | -3.445654 | -0.509012 | 2.404441 |
| C | -6.400205 | 0.369782 | 0.974489 |
| H | -6.829795 | 0.241959 | -1.129346 |
| H | -5.685968 | 0.360017 | 3.004887 |
| H | -7.378506 | 0.756977 | 1.234234 |
| C | -0.311078 | -5.482124 | -0.762531 |
| H | -0.372597 | -6.551581 | -0.926873 |
| C | -1.471834 | -4.728603 | -0.651775 |
| H | -2.444041 | -5.203471 | -0.721098 |

| **(*P,M*)-4c** | | | |
| --- | --- | --- | --- |
| Charge = 0, Multiplicity = 1 | | | |
| Number of imaginary frequencies = 0 | | | |
| Electronic Energy = -1461.87213786 Hartree | | | |
| Sum of electronic and thermal Free Energies = -1461.453630 Hartree | | | |
|  | | | |
| Atoms | Cartesian Coordinates | | |
|  | **X** | **Y** | **Z** |
| C | -1.222514 | -3.402140 | -0.581952 |
| C | -0.000315 | -2.685323 | -0.480735 |
| C | 1.221717 | -3.402439 | -0.581939 |
| C | 1.203384 | -4.779928 | -0.845400 |
| C | -2.430592 | -2.699508 | -0.310780 |
| C | -0.000155 | -1.264225 | -0.300573 |
| C | -1.247749 | -0.562639 | -0.229740 |
| C | -2.460443 | -1.354699 | -0.079209 |
| C | 1.247626 | -0.562990 | -0.229873 |
| C | 2.460140 | -1.355315 | -0.079198 |
| C | 2.429975 | -2.700121 | -0.310710 |
| H | 3.342202 | -3.274500 | -0.195962 |
| H | -3.343003 | -3.273627 | -0.196189 |
| H | 2.144893 | -5.311970 | -0.922797 |
| C | -1.244166 | 0.857629 | -0.340700 |
| C | 0.000251 | 1.560429 | -0.390363 |
| C | 1.244450 | 0.857241 | -0.341101 |
| C | -2.447328 | 1.623563 | -0.514075 |
| C | 0.000485 | 2.990688 | -0.512428 |
| C | -1.222516 | 3.716254 | -0.582922 |
| C | -2.441295 | 2.976052 | -0.612123 |
| C | -1.202175 | 5.115514 | -0.667765 |
| H | -2.144093 | 5.650500 | -0.716674 |
| C | 0.000951 | 5.809065 | -0.699816 |
| C | 1.203842 | 5.115070 | -0.668361 |
| C | 1.223723 | 3.715808 | -0.583513 |
| C | 2.442233 | 2.975174 | -0.613317 |
| C | 2.447820 | 1.622697 | -0.515149 |
| H | 3.375332 | 3.511839 | -0.745109 |
| H | -3.374282 | 3.513051 | -0.743355 |
| H | 0.001136 | 6.890976 | -0.764132 |
| H | 2.145928 | 5.649718 | -0.717751 |
| H | -3.390331 | 1.113461 | -0.594278 |
| H | 3.390575 | 1.112192 | -0.595810 |
| C | 3.735400 | -0.812945 | 0.464398 |
| C | 3.746724 | -0.163689 | 1.705506 |
| C | 4.948241 | -1.005218 | -0.204973 |
| C | 4.938731 | 0.292571 | 2.253847 |
| H | 2.811678 | -0.013345 | 2.231547 |
| C | 6.142521 | -0.543227 | 0.343044 |
| H | 4.947130 | -1.497380 | -1.170694 |
| C | 6.141804 | 0.109075 | 1.572778 |
| H | 4.930487 | 0.792279 | 3.215691 |
| H | 7.072535 | -0.689739 | -0.194290 |
| H | 7.070168 | 0.470669 | 1.999104 |
| C | -3.735723 | -0.812165 | 0.464218 |
| C | -4.948408 | -1.003810 | -0.205607 |
| C | -3.747226 | -0.163445 | 1.705602 |
| C | -6.142722 | -0.541702 | 0.342246 |
| H | -4.947140 | -1.495585 | -1.171524 |
| C | -4.939265 | 0.292941 | 2.253770 |
| H | -2.812288 | -0.013598 | 2.231981 |
| C | -6.142184 | 0.110086 | 1.572251 |
| H | -7.072619 | -0.687725 | -0.195424 |
| H | -4.931176 | 0.792231 | 3.215832 |
| H | -7.070571 | 0.471771 | 1.998449 |
| C | -0.000654 | -5.457729 | -0.991178 |
| H | -0.000779 | -6.521554 | -1.198169 |
| C | -1.204524 | -4.779636 | -0.845426 |
| H | -2.146165 | -5.311440 | -0.922854 |

| **(*P,P*)-4a (S_0_)** | | | |
| --- | --- | --- | --- |
| Charge = 0, Multiplicity = 1 | | | |
| Number of imaginary frequencies = 0 | | | |
| Electronic Energy = -1477.91252958 Hartree | | | |
| Sum of electronic and zero-point Energies = -1477.450221 Hartree | | | |
| Sum of electronic and thermal Free Energies = -1477.505450 Hartree | | | |
|  | | | |
| Atoms | Cartesian Coordinates | | |
|  | **X** | **Y** | **Z** |
| C | -1.209479 | 3.484562 | -0.083798 |
| C | -1.138850 | 4.886185 | -0.069742 |
| C | 1.139049 | 4.886140 | 0.069833 |
| C | 1.209623 | 3.484513 | 0.083864 |
| C | 0.000058 | 2.753996 | 0.000026 |
| C | 0.000031 | 1.328586 | 0.000012 |
| C | -1.240537 | 0.631923 | -0.156404 |
| C | -2.474420 | 1.398691 | -0.114102 |
| C | -2.436578 | 2.765626 | -0.105470 |
| C | 1.240571 | 0.631868 | 0.156417 |
| C | 1.192585 | -0.777722 | 0.351048 |
| C | -0.000032 | -1.479830 | 0.000007 |
| C | -1.192616 | -0.777668 | -0.351032 |
| C | 2.436693 | 2.765528 | 0.105516 |
| C | 2.474484 | 1.398591 | 0.114114 |
| C | -0.000068 | -2.911379 | 0.000012 |
| C | -1.127323 | -3.632801 | -0.485506 |
| C | -2.241886 | -2.890115 | -0.983107 |
| C | -2.277149 | -1.534921 | -0.906959 |
| C | 1.127148 | -3.632854 | 0.485538 |
| C | 1.102339 | -5.034649 | 0.486949 |
| C | -0.000138 | -5.726777 | 0.000018 |
| C | -1.102581 | -5.034597 | -0.486912 |
| C | 2.277068 | -1.535028 | 0.907004 |
| C | 2.241739 | -2.890220 | 0.983153 |
| C | -3.809494 | 0.765465 | 0.065670 |
| C | 3.809537 | 0.765334 | -0.065707 |
| C | -4.046420 | -0.087462 | 1.150772 |
| C | -5.304117 | -0.641793 | 1.352019 |
| C | -6.347605 | -0.355548 | 0.472316 |
| C | -6.124704 | 0.498005 | -0.604331 |
| C | -4.865166 | 1.058541 | -0.803321 |
| C | 4.865209 | 1.058282 | 0.803327 |
| C | 6.124730 | 0.497724 | 0.604292 |
| C | 6.347617 | -0.355717 | -0.472446 |
| C | 5.304132 | -0.641826 | -1.352197 |
| C | 4.046451 | -0.087476 | -1.150904 |
| H | -2.055965 | 5.467753 | -0.115545 |
| H | 2.056187 | 5.467670 | 0.115644 |
| H | -3.365449 | 3.320704 | -0.040095 |
| H | 3.365585 | 3.320571 | 0.040141 |
| H | -3.071453 | -3.430356 | -1.425425 |
| H | -3.134370 | -1.012878 | -1.299316 |
| H | 1.959966 | -5.574407 | 0.872757 |
| H | -0.000164 | -6.810543 | 0.000021 |
| H | -1.960237 | -5.574315 | -0.872713 |
| H | 3.134303 | -1.013027 | 1.299384 |
| H | 3.071271 | -3.430498 | 1.425490 |
| H | -3.235220 | -0.317248 | 1.830938 |
| H | -5.471705 | -1.298733 | 2.197628 |
| H | -7.326688 | -0.793377 | 0.627430 |
| H | -6.929696 | 0.726488 | -1.293362 |
| H | -4.687992 | 1.711004 | -1.650600 |
| H | 4.688046 | 1.710658 | 1.650675 |
| H | 6.929722 | 0.726104 | 1.293358 |
| H | 7.326687 | -0.793561 | -0.627597 |
| H | 5.471710 | -1.298675 | -2.197879 |
| H | 3.235253 | -0.317156 | -1.831108 |
| N | 0.000113 | 5.575249 | 0.000052 |

| **(*P,P*)-4a (TS)** | | | |
| --- | --- | --- | --- |
| Charge = 0, Multiplicity = 1 | | | |
| Number of imaginary frequencies = 1, *v*_i_ = -22.64 | | | |
| Electronic Energy = -1477.90783016 Hartree | | | |
| Sum of electronic and thermal Free Energies = -1477.499563 Hartree | | | |
|  | | | |
| Atoms | Cartesian Coordinates | | |
|  | **X** | **Y** | **Z** |
| C | 1.035399 | -3.472824 | -0.432700 |
| C | 0.891882 | -4.854555 | -0.626215 |
| C | -1.384184 | -4.734735 | -0.631527 |
| C | -1.377862 | -3.346055 | -0.427290 |
| C | -0.135111 | -2.679686 | -0.347411 |
| C | -0.066197 | -1.259785 | -0.204438 |
| C | 1.225537 | -0.643774 | -0.208559 |
| C | 2.401924 | -1.493098 | -0.069265 |
| C | 2.294090 | -2.844868 | -0.227121 |
| C | -1.275731 | -0.489687 | -0.098340 |
| C | -1.169041 | 0.938168 | -0.088441 |
| C | 0.114284 | 1.550556 | -0.276572 |
| C | 1.301848 | 0.763897 | -0.381098 |
| C | -2.558662 | -2.580039 | -0.245120 |
| C | -2.532182 | -1.229295 | -0.041015 |
| C | 0.219178 | 2.979414 | -0.375337 |
| C | 1.472228 | 3.604062 | -0.640222 |
| C | 2.609388 | 2.771783 | -0.862754 |
| C | 2.524362 | 1.424719 | -0.742387 |
| C | -0.928525 | 3.806010 | -0.222060 |
| C | -0.805037 | 5.201164 | -0.295360 |
| C | 0.427176 | 5.793126 | -0.536854 |
| C | 1.554599 | 5.001030 | -0.716816 |
| C | -2.297615 | 1.818291 | 0.033936 |
| C | -2.184885 | 3.169796 | -0.012836 |
| C | 3.720458 | -0.976616 | 0.388572 |
| C | -3.867540 | -0.645723 | 0.291499 |
| C | 3.808093 | -0.213788 | 1.560086 |
| C | 5.040375 | 0.225756 | 2.026795 |
| C | 6.207909 | -0.087133 | 1.331607 |
| C | 6.132657 | -0.851437 | 0.170550 |
| C | 4.898107 | -1.296685 | -0.295205 |
| C | -4.192873 | -0.361129 | 1.621942 |
| C | -5.452837 | 0.127170 | 1.950535 |
| C | -6.407351 | 0.329539 | 0.954645 |
| C | -6.097643 | 0.028536 | -0.368980 |
| C | -4.836113 | -0.462225 | -0.698233 |
| H | 1.779353 | -5.478850 | -0.689217 |
| H | -2.333299 | -5.259048 | -0.706544 |
| H | 3.173244 | -3.467784 | -0.109017 |
| H | -3.512546 | -3.094387 | -0.217562 |
| H | 3.547807 | 3.234454 | -1.147262 |
| H | 3.397506 | 0.832008 | -0.953847 |
| H | -1.691348 | 5.812246 | -0.166383 |
| H | 0.508579 | 6.872279 | -0.594302 |
| H | 2.516227 | 5.457222 | -0.923740 |
| H | -3.279077 | 1.409109 | 0.166447 |
| H | -3.070838 | 3.786300 | 0.092831 |
| H | 2.901725 | 0.036295 | 2.097953 |
| H | 5.091154 | 0.814034 | 2.935703 |
| H | 7.167883 | 0.261714 | 1.693649 |
| H | 7.034734 | -1.098465 | -0.377348 |
| H | 4.838954 | -1.876226 | -1.209252 |
| H | -3.446051 | -0.512183 | 2.392589 |
| H | -5.692054 | 0.346903 | 2.984623 |
| H | -7.388347 | 0.712082 | 1.210902 |
| H | -6.837705 | 0.175212 | -1.147246 |
| H | -4.588261 | -0.687615 | -1.728794 |
| N | -0.282607 | -5.476372 | -0.736035 |

| **(*P,M*)-4a** | | | |
| --- | --- | --- | --- |
| Charge = 0, Multiplicity = 1 | | | |
| Number of imaginary frequencies = 0 | | | |
| Electronic Energy = -1477.90876546 Hartree | | | |
| Sum of electronic and thermal Free Energies = -1477.501836 Hartree | | | |
|  | | | |
| Atoms | Cartesian Coordinates | | |
|  | **X** | **Y** | **Z** |
| C | 1.209729 | -3.398418 | -0.565212 |
| C | 1.140162 | -4.777866 | -0.812387 |
| C | -1.140705 | -4.777736 | -0.812400 |
| C | -1.210113 | -3.398279 | -0.565220 |
| C | -0.000153 | -2.672551 | -0.465313 |
| C | -0.000077 | -1.255123 | -0.289418 |
| C | 1.251465 | -0.562512 | -0.224814 |
| C | 2.463172 | -1.363052 | -0.085158 |
| C | 2.427959 | -2.709707 | -0.312160 |
| C | -1.251532 | -0.562345 | -0.224762 |
| C | -1.246888 | 0.858362 | -0.330009 |
| C | 0.000117 | 1.559699 | -0.374415 |
| C | 1.247017 | 0.858176 | -0.330185 |
| C | -2.428260 | -2.709418 | -0.312194 |
| C | -2.463324 | -1.362760 | -0.085170 |
| C | 0.000231 | 2.990911 | -0.487984 |
| C | 1.223184 | 3.717044 | -0.554888 |
| C | 2.442385 | 2.978282 | -0.590146 |
| C | 2.449468 | 1.625404 | -0.500597 |
| C | -1.222607 | 3.717257 | -0.554621 |
| C | -1.202424 | 5.116960 | -0.630620 |
| C | 0.000457 | 5.810940 | -0.657928 |
| C | 1.203225 | 5.116747 | -0.630889 |
| C | -2.449236 | 1.625819 | -0.500114 |
| C | -2.441936 | 2.978702 | -0.589607 |
| C | 3.746972 | -0.823181 | 0.441147 |
| C | -3.747130 | -0.822811 | 0.441062 |
| C | 3.777651 | -0.183925 | 1.687011 |
| C | 4.978095 | 0.269970 | 2.218635 |
| C | 6.169532 | 0.093787 | 1.515558 |
| C | 6.150772 | -0.549295 | 0.281148 |
| C | 4.948182 | -1.009268 | -0.250219 |
| C | -3.777885 | -0.183802 | 1.687050 |
| C | -4.978338 | 0.270159 | 2.218600 |
| C | -6.169703 | 0.094277 | 1.515325 |
| C | -6.150868 | -0.548572 | 0.280795 |
| C | -4.948269 | -1.008607 | -0.250501 |
| H | 2.058927 | -5.352356 | -0.896849 |
| H | -2.059537 | -5.352117 | -0.896878 |
| H | 3.338477 | -3.288160 | -0.205437 |
| H | -3.338864 | -3.287748 | -0.205542 |
| H | 3.374781 | 3.516835 | -0.718544 |
| H | 3.392405 | 1.115976 | -0.583922 |
| H | -2.144386 | 5.651977 | -0.676621 |
| H | 0.000547 | 6.893148 | -0.715452 |
| H | 2.145269 | 5.651603 | -0.677107 |
| H | -3.392291 | 1.116584 | -0.583237 |
| H | -3.374277 | 3.517415 | -0.717752 |
| H | 2.851618 | -0.039638 | 2.230498 |
| H | 4.985645 | 0.761506 | 3.184623 |
| H | 7.104443 | 0.453287 | 1.929035 |
| H | 7.071868 | -0.690859 | -0.272452 |
| H | 4.931660 | -1.495067 | -1.218995 |
| H | -2.851902 | -0.039747 | 2.230685 |
| H | -4.985953 | 0.761504 | 3.184684 |
| H | -7.104621 | 0.453825 | 1.928746 |
| H | -7.071911 | -0.689908 | -0.272951 |
| H | -4.931680 | -1.494232 | -1.219363 |
| N | -0.000311 | -5.454776 | -0.946221 |

| **(*P,P*)-4b (S_0_)** | | | |
| --- | --- | --- | --- |
| Charge = 0, Multiplicity = 1 | | | |
| Number of imaginary frequencies = 0 | | | |
| Electronic Energy = -1477.91508443 Hartree | | | |
| Sum of electronic and zero-point Energies = -1477.452689 Hartree | | | |
| Sum of electronic and thermal Free Energies = -1477.507882 Hartree | | | |
|  | | | |
| Atoms | Cartesian Coordinates | | |
|  | **X** | **Y** | **Z** |
| C | 1.200375 | -3.495954 | -0.085538 |
| C | 1.120133 | -4.894429 | -0.071521 |
| C | -0.128186 | -5.499905 | 0.014372 |
| C | -1.225102 | -3.488489 | 0.087224 |
| C | -0.008213 | -2.760864 | 0.000407 |
| C | -0.003655 | -1.334628 | -0.001000 |
| C | 1.238255 | -0.639607 | -0.157448 |
| C | 2.467950 | -1.410030 | -0.116590 |
| C | 2.428354 | -2.777069 | -0.109086 |
| C | -1.239026 | -0.629242 | 0.152949 |
| C | -1.184331 | 0.780198 | 0.349284 |
| C | 0.010369 | 1.478757 | 0.000238 |
| C | 1.198892 | 0.770555 | -0.350813 |
| C | -2.445681 | -2.754952 | 0.106357 |
| C | -2.475831 | -1.388490 | 0.111365 |
| C | 0.016857 | 2.909881 | 0.002349 |
| C | 1.146929 | 3.626899 | -0.482252 |
| C | 2.257354 | 2.879251 | -0.981333 |
| C | 2.286323 | 1.523607 | -0.907336 |
| C | -1.106879 | 3.635441 | 0.488986 |
| C | -1.075909 | 5.037113 | 0.492551 |
| C | 0.029312 | 5.725411 | 0.006234 |
| C | 1.128452 | 5.028789 | -0.482008 |
| C | -2.264998 | 1.541199 | 0.908018 |
| C | -2.223924 | 2.896306 | 0.985918 |
| C | 3.805515 | -0.782155 | 0.062706 |
| C | -3.807552 | -0.748622 | -0.069701 |
| C | 4.045510 | 0.067811 | 1.149308 |
| C | 5.305406 | 0.617020 | 1.350887 |
| C | 6.346900 | 0.329354 | 0.469363 |
| C | 6.120485 | -0.521303 | -0.608931 |
| C | 4.859328 | -1.078163 | -0.807436 |
| C | -4.867080 | -1.042415 | 0.794220 |
| C | -6.122812 | -0.473755 | 0.595433 |
| C | -6.338558 | 0.387498 | -0.476696 |
| C | -5.291708 | 0.672978 | -1.352477 |
| C | -4.037290 | 0.111224 | -1.150833 |
| H | 2.020430 | -5.495526 | -0.121894 |
| H | -0.206254 | -6.583586 | 0.024067 |
| H | 3.356262 | -3.333148 | -0.043001 |
| H | -3.362623 | -3.326796 | 0.044434 |
| H | 3.089200 | 3.415826 | -1.423933 |
| H | 3.140493 | 0.998708 | -1.302439 |
| H | -1.931058 | 5.579984 | 0.879541 |
| H | 0.034018 | 6.809127 | 0.007653 |
| H | 1.988280 | 5.565171 | -0.867707 |
| H | -3.123987 | 1.022047 | 1.300247 |
| H | -3.051244 | 3.438982 | 1.429532 |
| H | 3.235292 | 0.299054 | 1.830053 |
| H | 5.475970 | 1.271631 | 2.197668 |
| H | 7.327484 | 0.763850 | 0.624344 |
| H | 6.924140 | -0.750636 | -1.299209 |
| H | 4.679282 | -1.728993 | -1.655323 |
| H | -4.696146 | -1.704204 | 1.635350 |
| H | -6.931145 | -0.703261 | 1.280196 |
| H | -7.315175 | 0.830834 | -0.631969 |
| H | -5.454056 | 1.334698 | -2.195413 |
| H | -3.223514 | 0.339603 | -1.828401 |
| N | -1.281634 | -4.834095 | 0.087597 |

| **(*P,P*)-4b (TS)** | | | |
| --- | --- | --- | --- |
| Charge = 0, Multiplicity = 1 | | | |
| Number of imaginary frequencies = 1, *v*_i_ = -23.31 | | | |
| Electronic Energy = -1477.91041478 Hartree | | | |
| Sum of electronic and thermal Free Energies = -1477.502032 Hartree | | | |
|  | | | |
| Atoms | Cartesian Coordinates | | |
|  | **X** | **Y** | **Z** |
| C | 1.022346 | -3.486336 | -0.430217 |
| C | 0.867757 | -4.863656 | -0.628729 |
| C | -0.413100 | -5.393675 | -0.733614 |
| C | -1.397453 | -3.347945 | -0.429192 |
| C | -0.146373 | -2.687192 | -0.348055 |
| C | -0.071025 | -1.266841 | -0.205830 |
| C | 1.222716 | -0.654589 | -0.210208 |
| C | 2.393285 | -1.508055 | -0.066673 |
| C | 2.282028 | -2.860296 | -0.219623 |
| C | -1.272966 | -0.486588 | -0.093508 |
| C | -1.156597 | 0.941030 | -0.079558 |
| C | 0.128082 | 1.546915 | -0.276149 |
| C | 1.309133 | 0.752024 | -0.385534 |
| C | -2.569425 | -2.566469 | -0.241730 |
| C | -2.533018 | -1.216874 | -0.035962 |
| C | 0.241607 | 2.974574 | -0.376146 |
| C | 1.496372 | 3.590869 | -0.650848 |
| C | 2.626484 | 2.750645 | -0.879290 |
| C | 2.533880 | 1.404219 | -0.755495 |
| C | -0.899209 | 3.808296 | -0.213474 |
| C | -0.767652 | 5.202626 | -0.289255 |
| C | 0.466001 | 5.786807 | -0.542226 |
| C | 1.586893 | 4.987147 | -0.730293 |
| C | -2.277799 | 1.828455 | 0.057492 |
| C | -2.156680 | 3.179459 | 0.009854 |
| C | 3.713023 | -0.995421 | 0.391509 |
| C | -3.866087 | -0.624962 | 0.291963 |
| C | 3.800289 | -0.224939 | 1.557923 |
| C | 5.033411 | 0.211484 | 2.025366 |
| C | 6.201191 | -0.111093 | 1.335139 |
| C | 6.125896 | -0.882783 | 0.178907 |
| C | 4.890837 | -1.326462 | -0.286687 |
| C | -4.200445 | -0.352326 | 1.622410 |
| C | -5.458351 | 0.145412 | 1.945186 |
| C | -6.401275 | 0.369261 | 0.943016 |
| C | -6.082612 | 0.079267 | -0.381047 |
| C | -4.823570 | -0.421302 | -0.704359 |
| H | 1.736820 | -5.507854 | -0.692624 |
| H | -0.549389 | -6.458945 | -0.898714 |
| H | 3.158166 | -3.485568 | -0.094155 |
| H | -3.513637 | -3.095325 | -0.220890 |
| H | 3.566025 | 3.206583 | -1.171013 |
| H | 3.401889 | 0.805634 | -0.971905 |
| H | -1.648907 | 5.819384 | -0.152695 |
| H | 0.553671 | 6.865319 | -0.601764 |
| H | 2.549702 | 5.437062 | -0.945462 |
| H | -3.259991 | 1.425535 | 0.202779 |
| H | -3.037606 | 3.801170 | 0.126776 |
| H | 2.893437 | 0.033050 | 2.091049 |
| H | 5.084463 | 0.805518 | 2.930464 |
| H | 7.161723 | 0.236057 | 1.697330 |
| H | 7.028381 | -1.137055 | -0.364947 |
| H | 4.831446 | -1.912365 | -1.196619 |
| H | -3.462675 | -0.521048 | 2.398085 |
| H | -5.705060 | 0.355130 | 2.979618 |
| H | -7.380780 | 0.758713 | 1.194640 |
| H | -6.814451 | 0.240835 | -1.164134 |
| H | -4.569601 | -0.641274 | -1.734515 |
| N | -1.529883 | -4.673524 | -0.628811 |
| **(*P,M*)-4b** | | | |
| Charge = 0, Multiplicity = 1 | | | |
| Number of imaginary frequencies = 0 | | | |
| Electronic Energy = -1477.91151259 Hartree | | | |
| Sum of electronic and thermal Free Energies = -1477.504508 Hartree | | | |
|  | | | |
| Atoms | Cartesian Coordinates | | |
|  | X | Y | Z |
| C | 1.201032 | -3.409474 | -0.573686 |
| C | 1.121898 | -4.783779 | -0.832252 |
| C | -0.128939 | -5.375163 | -0.964484 |
| C | -1.225850 | -3.400138 | -0.580648 |
| C | -0.007948 | -2.678623 | -0.476934 |
| C | -0.002857 | -1.261180 | -0.298463 |
| C | 1.249656 | -0.570427 | -0.229141 |
| C | 2.455515 | -1.375285 | -0.082957 |
| C | 2.418764 | -2.722249 | -0.309687 |
| C | -1.248533 | -0.560112 | -0.226623 |
| C | -1.237861 | 0.860448 | -0.335116 |
| C | 0.010747 | 1.557910 | -0.381470 |
| C | 1.253732 | 0.850350 | -0.333874 |
| C | -2.436126 | -2.698819 | -0.313960 |
| C | -2.462309 | -1.353547 | -0.081136 |
| C | 0.016499 | 2.988374 | -0.498617 |
| C | 1.242061 | 3.709509 | -0.565407 |
| C | 2.458051 | 2.965351 | -0.595707 |
| C | 2.459778 | 1.612456 | -0.503015 |
| C | -1.203349 | 3.718852 | -0.568929 |
| C | -1.177831 | 5.118215 | -0.649427 |
| C | 0.027604 | 5.807886 | -0.677174 |
| C | 1.227645 | 5.109046 | -0.645878 |
| C | -2.437529 | 1.631478 | -0.508924 |
| C | -2.425023 | 2.984184 | -0.602882 |
| C | 3.738005 | -0.840327 | 0.451136 |
| C | -3.739063 | -0.807091 | 0.454896 |
| C | 3.761868 | -0.199296 | 1.696099 |
| C | 4.960482 | 0.250480 | 2.235332 |
| C | 6.155760 | 0.069477 | 1.540134 |
| C | 6.143244 | -0.575103 | 0.306360 |
| C | 4.942880 | -1.032549 | -0.231969 |
| C | -3.754931 | -0.154980 | 1.694297 |
| C | -4.948715 | 0.306655 | 2.234361 |
| C | -6.147761 | 0.126011 | 1.545699 |
| C | -6.143464 | -0.530139 | 0.317890 |
| C | -4.947943 | -0.998745 | -0.221307 |
| H | 2.023805 | -5.378453 | -0.917042 |
| H | -0.208197 | -6.438158 | -1.174930 |
| H | 3.326513 | -3.302955 | -0.194534 |
| H | -3.335044 | -3.293244 | -0.212807 |
| H | 3.393069 | 3.499598 | -0.723411 |
| H | 3.401109 | 1.099617 | -0.583873 |
| H | -2.117767 | 5.656562 | -0.698352 |
| H | 0.031804 | 6.889863 | -0.738151 |
| H | 2.171814 | 5.640216 | -0.691993 |
| H | -3.382063 | 1.124686 | -0.591485 |
| H | -3.355432 | 3.525593 | -0.733896 |
| H | 2.832503 | -0.050668 | 2.232532 |
| H | 4.963384 | 0.743271 | 3.200667 |
| H | 7.089093 | 0.426553 | 1.959233 |
| H | 7.067529 | -0.720074 | -0.240970 |
| H | 4.930987 | -1.520109 | -1.199892 |
| H | -2.822877 | -0.007721 | 2.226556 |
| H | -4.944975 | 0.807612 | 3.195546 |
| H | -7.077607 | 0.491363 | 1.965513 |
| H | -7.070887 | -0.676651 | -0.223728 |
| H | -4.942935 | -1.498464 | -1.182947 |
| N | -1.284137 | -4.722340 | -0.827322 |

# **NMR Spectra**

**Figure S10**. ^1^H-NMR spectrum of **2c** in CDCl_3_ at 298 K.

**Figure S11.** ^13^C{^1^H}-NMR spectrum of **2c** in CDCl_3_ at 298 K.

**Figure S12**. ^1^H-NMR spectrum of **2d** in CDCl_3_ at 298 K.

**Figure S13.** ^13^C{^1^H}-NMR spectrum of **2d** in CDCl_3_ at 298 K.

**Figure S14**. ^1^H-NMR spectrum of **2e** in CDCl_3_ at 298 K.

**Figure S15.** ^13^C{^1^H}-NMR spectrum of **2e** in CDCl_3_ at 298 K.

**Figure S16**. ^1^H-NMR spectrum of **2f** in CDCl_3_ at 298 K.

**Figure S17.** ^13^C{^1^H}-NMR spectrum of **2f** in CDCl_3_ at 298 K. **Figure S18**. ^1^H-NMR spectrum of **3a** in CDCl_3_ at 298 K.

**Figure S19.** ^13^C{^1^H}-NMR spectrum of **3a** in CDCl_3_ at 298 K.

**Figure S20**. ^1^H-NMR spectrum of **3b** in CDCl_3_ at 298 K.

**Figure S21.** ^13^C{^1^H}-NMR spectrum of **3b** in CDCl_3_ at 298 K.

**Figure S22**. ^1^H-NMR spectrum of **3c** in CDCl_3_ at 298 K.

**Figure S23.** ^13^C{^1^H}-NMR spectrum of **3c** in CDCl_3_ at 298 K.

**Figure S24.** Partial ^1^H-^13^C{^1^H} HSQC-DEPT spectrum of **3c** in CDCl_3_ at 298 K.

**Figure S25**. ^1^H-NMR spectrum of **4a** in CDCl_3_ at 298 K.

**Figure S26.** ^13^C{^1^H}-NMR spectrum of **4a** in CDCl_3_ at 298 K.

**Figure S27**. ^1^H-NMR spectrum of **4b** in CDCl_3_ at 298 K.

**Figure S28.** ^13^C{^1^H}-NMR spectrum of **4b** in CDCl_3_ at 298 K.

**Figure S29**. ^1^H-NMR spectrum of **4c** in CDCl_3_ at 298 K.

**Figure S30.** ^13^C{^1^H}-NMR spectrum of **4c** in CDCl_3_ at 298 K.

# **References**

[1] K. H. Drexhage, *J. RES. NATL. BUR. STAN. SECT. A.* **1976**, *80A*, 421.

[2] R. Molenda, S. Boldt, A. Villinger, P. Ehlers, P. Langer, *J. Org. Chem.* **2020**, *85*, 12823–12842.

[3] A. Vardanyan, S. Boldt, A. Villinger, P. Ehlers, P. Langer, *J. Org. Chem.* **2022**, *87*, 11296–11308.

[4] E. Marzi, A. Bigi, M. Schlosser, *Eur. J. Org. Chem.* **2001**, 1371–1376.

[5] T. Dumslaff, B. Yang, A. Maghsoumi, G. Velpula, K. S. Mali, C. Castiglioni,S. De Feyter, M. Tommasini, A. Narita, X. Feng, K. Müllen, *J. Am. Chem. Soc.* **2016**, *138*, 4726–4729.

[6] M. Schlosser, C. Heiss, E. Marzi; R. Scopelliti, *Eur. J. Org. Chem.* **2006**, 4398–4404.

[7] V. M. Nichols, M. T. Rodriguez, G. B. Piland, F. Tham, V. N. Nesterov, W. J. Youngblood, C. J. Bardeen *J. Phys. Chem. C* **2013**, *117*, 16802−16810.

[8] U. Wenzel, H. G. Lohmannsroben, *J. Photochem. Photobiol., A* **1996**, *96*, 13–18.

[9] E. M. Espinoza, J. A. Clark, J. Soliman, J. B. Derr, M. Morales, V. I. Vullev, *J. Electrochem. Soc.* **2019**, *166*, 3175-3187.

[10] Gaussian 09, Revision E.01, M. J. Frisch, G. W. Trucks, H. B. Schlegel, G. E. Scuseria, M. A. Robb, J. R. Cheeseman, G. Scalmani, V. Barone, B. Mennucci, G. A. Petersson, H. Nakatsuji, M. Caricato, X. Li, H. P. Hratchian, A. F. Izmaylov, J. Bloino, G. Zheng, J. L. Sonnenberg, M. Hada, M. Ehara, K. Toyota, R. Fukuda, J. Hasegawa, M. Ishida, T. Nakajima, Y. Honda, O. Kitao, H. Nakai, T. Vreven, J. A. Montgomery, Jr., J. E. Peralta, F. Ogliaro, M. Bearpark, J. J. Heyd, E. Brothers, K. N. Kudin, V. N. Staroverov, T. Keith, R. Kobayashi, J. Normand, K. Raghavachari, A. Rendell, J. C. Burant, S. S. Iyengar, J. Tomasi, M. Cossi, N. Rega, J. M. Millam, M. Klene, J. E. Knox, J. B. Cross, V. Bakken, C. Adamo, J. Jaramillo, R. Gomperts, R. E. Stratmann, O. Yazyev, A. J. Austin, R. Cammi, C. Pomelli, J. W. Ochterski, R. L. Martin, K. Morokuma, V. G. Zakrzewski, G. A. Voth, P. Salvador, J. J. Dannenberg, S. Dapprich, A. D. Daniels, O. Farkas, J. B. Foresman, J. V. Ortiz, J. Cioslowski, and D. J. Fox, Gaussian, Inc., Wallingford CT, **2013**.

[11] a) C. Lee, W. Yang W., R.G. Parr, *Phys. Rev. B* **1988,** *37*, 785-789; b) A.D. Becke, *Chem. Phys.* **1993**, *98*, 5648-5652.

[12] a) R. Ditchfield, W. J. Hehre, J. A. Pople, *J. Chem. Phys.* **1971**, *54*, 724-728; b) W. J. Hehre, R. Ditchfield, J. A. Pople, *J. Chem. Phys.* **1972**, *56*, 2257-2261.

[13] S. Grimme, J. Antony, S. Ehrlich, H. Krieg, *J. Chem. Phys*. **2010**, *132*, 154104.

[14] S. Grimme, S. Ehrlich, L. Goerigk, *J. Comput. Chem*. **2011**, *32*, 1456–1465.

[15] S. Hiarta, M. Head-Gordon, *Chem. Phys. Lett.* **1999**, *314*, 291-299.

[16] a) P.W. Fowler, P. Lazzeretti, R. Zanasi, *Chem. Phys. Lett*. **1990**, *165*, 79-86; b) R. Zanasi, P.W. Fowler, *Chem. Phys. Lett*. **1995**, *238*, 270-280; c) P. V. R. Schleyer, C. Maerker, A. Dransfeld, H. Jiao, N. J.R. van Eikema Hommes, *J. Am. Chem. Soc*. **1996**, *118*, 6317-6318.

[17] T. Lu, *J. Chem. Phys.* **2024**, *161*, 082503.

[18] a) A. Stanger, *J. Org. Chem*. **2010**, *75*, 2281−2288; b) R. Gershoni-Poranne, A. Stanger, *Chem. - Eur. J.* **2014**, *20*, 5673– 5688.

[19] E. Paenurk, R. Gershoni-Poranne, *Phys. Chem. Chem. Phys.* **2022**, 24, 8631– 8644.

[20] a) R. Herges, D. Geuenich, *J. Phys. Chem.* *A* **2001**, *105*, 3214–3220; b) D. Geuenich, K. Hess, F. Köhler, R. Herges, *Chem. Rev*. **2015**, *105*, 3758–3772.

[21] T. Lu, Q. Chen, *Theo. Chem. Acc.* **2020**, 139:25.

[22] W. Humphrey, A. Dalke, K. Schulten, *J. Mol. Graph*. **1996**, *14*, 33-38.
